# Grainyhead-like 2 interacts with noggin to regulate tissue fusion in mouse

**DOI:** 10.1242/dev.202420

**Published:** 2024-02-28

**Authors:** Michael E. de Vries, Marina R. Carpinelli, Jarrad N. Fuller, Yindi Sutton, Darren D. Partridge, Alana Auden, Peter J. Anderson, Stephen M. Jane, Sebastian Dworkin

**Affiliations:** ^1^ Department of Medicine, Monash University Central Clinical School, Prahran, Victoria 3004, Australia; ^2^ Department of Physiology, Anatomy and Microbiology, La Trobe University, Melbourne, Victoria 3086, Australia; ^3^ Australian Craniofacial Unit, Women and Children's Hospital, Adelaide, SA 5005, Australia; ^4^ Faculty of Health Sciences, University of Adelaide, Adelaide, SA 5005, Australia; ^5^ School of Basic Medical Sciences, Nanjing Medical University, Nanjing, 211166, People's Republic of China

**Keywords:** Craniofacial development, Transcription factor, Epithelia, Cleft lip/palate, Neural tube defects, Grhl2, Mouse

## Abstract

Defective tissue fusion during mammalian embryogenesis results in congenital anomalies, such as exencephaly, spina bifida and cleft lip and/or palate. The highly conserved transcription factor grainyhead-like 2 (Grhl2) is a crucial regulator of tissue fusion, with mouse models lacking GRHL2 function presenting with a fully penetrant open cranial neural tube, facial and abdominal clefting (abdominoschisis), and an open posterior neuropore. Here, we show that GRHL2 interacts with the soluble morphogen protein and bone morphogenetic protein (BMP) inhibitor noggin (NOG) to impact tissue fusion during development. The maxillary prominence epithelium in embryos lacking *Grhl2* shows substantial morphological abnormalities and significant upregulation of NOG expression, together with aberrantly distributed pSMAD5-positive cells within the neural crest cell-derived maxillary prominence mesenchyme, indicative of disrupted BMP signalling. Reducing this elevated NOG expression (by generating *Grhl2^−/−^;Nog^+/−^* embryos) results in delayed embryonic lethality, partial tissue fusion rescue, and restoration of tissue form within the craniofacial epithelia. These data suggest that aberrant epithelial maintenance, partially regulated by noggin-mediated regulation of BMP-SMAD pathways, may underpin tissue fusion defects in *Grhl2^−/−^* mice.

## Teaser

**Summary:** Genetic reduction of elevated noggin expression in *Grhl2^−/−^* embryos leads to rescue of epithelial morphology and partially restores maxillary prominence size and neural tube closure.

## INTRODUCTION

The grainyhead-like (GRHL) transcription factors (GRHL1-3) regulate craniofacial skeleton formation (Carpinelli et al., 2017). *GRHL3* mutations lead to both syndromic (Van der Woude Syndrome, VWS; Peyrard-Janvid et al., 2014) and non-syndromic (Leslie et al., 2016) palatal clefts, and craniofacial defects are also seen in *Grhl3^−/−^* mice (Goldie et al., 2016; Peyrard-Janvid et al., 2014). However, loss of the murine orthologue *Grhl2* results in far more severe defects, namely lethality by embryonic day (E) 11.5, fully penetrant open cranial and posterior neural tube (NT), ‘split-face’ (Rifat et al., 2010), including maxillary clefting (Pyrgaki et al., 2011), and craniofacial epithelia defects (Carpinelli et al., 2020). Genetic deletion of a *Grhl2* enhancer also contributes to palatal clefting (de Vries et al., 2020), strongly implicating *Grhl2* in mammalian craniofacial development.

The phenotype of *Grhl2^tm1.1Jane^* nullizygous embryos has been thoroughly described elsewhere (Carpinelli et al., 2020; Rifat et al., 2010), but, briefly, epithelia overlying palatal-shelf mesenchyme is substantially thicker, owing to an excess of cells with characteristics intermediate between those of epithelial and mesenchymal tissue (Carpinelli et al., 2020). As *Grhl2* is only expressed within the epithelial layer of the craniofacial primordia (Carpinelli et al., 2020), *Grhl2*-dependent mechanisms must exist within this layer to drive epithelial stability.

As transcription factors, the products of GRHL genes regulate multiple downstream targets. We had previously identified 305 genes that contain a conserved GRHL-binding site (broadly AA**C**CG**G**TT; bold and underlined nucleotides are invariant within the GRHL-binding site within target genes), and this dataset has proven a rich source of experimentally validated targets, including *Tgm1*, *Tgm5*, *Dsg1*, *ARHGEF19*, *PTEN*, *en2a*, *spec1* (*cdc42se1*), *edn1* and *GSK3B* (Caddy et al., 2010; Carpinelli et al., 2017; Darido et al., 2011; Dworkin et al., 2012, 2014; Gasperoni et al., 2022; Miles et al., 2017).

Here, we report characterisation of a novel target, noggin (NOG), that directly co-operates with GRHL2 in maintaining epithelial integrity during tissue fusion.

## RESULTS AND DISCUSSION

### *Nog* is a GRHL2 target gene within the murine craniofacial epithelium

We cross-referenced our previously published first pharyngeal arch (PA1) RNA-sequencing data from *Grhl2^−/−^* and *Grhl2^+/+^* control littermate mouse embryos (Carpinelli et al., 2020) with our list of 305 predicted GRHL targets. From this, we identified 21 differentially regulated genes, including the gene encoding the soluble morphogen NOG, a negative regulator of the BMP pathway that was significantly upregulated in *Grhl2^−/−^* PA1 (Fig. S1). Given the consequences of BMP loss on craniofacial development and neurulation (Kouskoura et al., 2013; Parada and Chai, 2012), we hypothesised that aberrant NOG expression underpins *Grhl2^−/−^* craniofacial and NT-fusion defects.

**Fig. 1.**
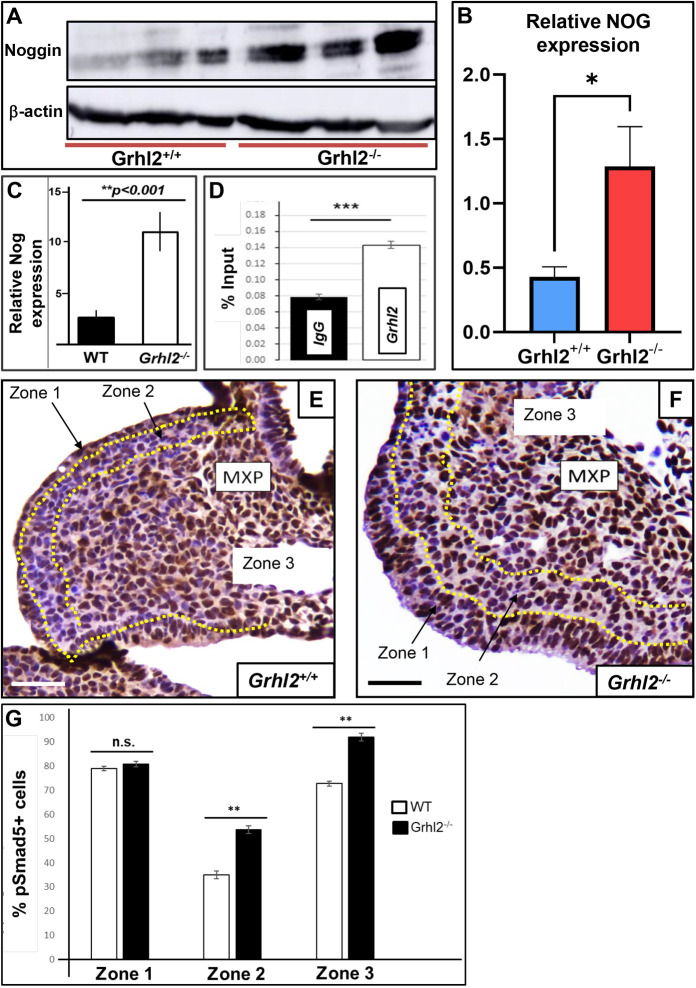
**Increased noggin expression correlates with aberrant pSMAD1/5/9 distribution in the maxillary prominence of *Grhl2^−/−^* embryos.** (A,B) Western blotting confirms ∼3-fold elevated noggin protein expression in whole *Grhl2^−/−^* E10.5 embryos relative to expression of β-actin by densitometric quantitation. *n*=3. (C) Q-RT-PCR of first pharyngeal arch (PA1) epithelium at E10.5 confirms ∼5-fold *Nog* upregulation in *Grhl2^−/−^* PA1 tissue. Relative expression for WT PA1 is 2.45±0.55 (*n*=6) and 12.07±1.96 (*n*=9) for *Grhl2^−/−^* PA1. (D) ChIP Q-RT-PCR analysis confirms ∼3-fold enrichment of the *Nog* promoter region in samples incubated with GRHL2 antibody relative to IgG negative control in whole E8.5 PA1 (*n*=3, *P*=0.00015). (E,F) pSMAD5 localisation (brown) in *Grhl2^+/+^* and *Grhl2^−/−^* E10.5 MXP coronal sections exhibited differential staining patterns in *Grhl2^−/−^* tissue relative to control littermates (*n*=3). The section was divided into three zones for characterisation of pSMAD5 distribution based on distance from the epithelium: Zone 1, ectodermal epithelial layer of MXP bounded by basement membrane; Zone 2, one to five cells' depth of mesenchyme extending from basement membrane; Zone 3, remaining PA1 mesenchymal cells extending from Zone 2 towards the oral/nasal cavity. Dotted yellow lines define the zone boundaries. (G) pSMAD5^+^ quantitation confirms a significantly greater cell number present in Zones 2 (*P*=0.01) and 3 (*P*=0.006) of *Grhl2^−/−^* MXP (*n*=3) than control littermates (*n*=3); no significant differences are seen within Zone 1 (*P*=0.25). Error bars indicate s.e.m. *P≤0.05, **P≤0.01, ***P≤0.001 (unpaired, two-tailed Student's *t*-test). n.s., not significant. Scale bars: 100 µm.

NOG characterisation at E10.5 confirmed significant protein overexpression in whole *Grhl2^−/−^* embryos (Fig. 1A,B). Next, we extracted maxillary prominence (MXP) epithelial mRNA (Carpinelli et al., 2020; Li and Williams, 2013) from wild-type (WT) (*n*=6) and *Grhl2^−/−^* (*n*=9) PA1, and, strikingly, found that *Nog* expression was ∼5-fold higher in *Grhl2^−/−^* PA1 (12.07±1.96; mean±s.e.m.) than WT PA1 (2.45±0.55; Fig. 1C), confirming our RNA-sequencing data in this refined cell population. In WT mice, *Nog* is expressed only in the palatal shelf mesenchyme (and not epithelium) at E8.5, before becoming expressed in the epithelium from ∼E10.5 onwards (Matsui and Klingensmith, 2014). This led us to hypothesise that GRHL2 directly represses *Nog* in the epithelium at E8.5. Therefore, we performed chromatin immunoprecipitation (ChIP) analysis from E8.5 PA1 tissue, and interrogated GRHL2 binding at the predicted binding site [AACTAGTT; chr11:89,289,343-89,289,336; 11,295 kb upstream of the transcriptional start site (TSS)] in the *Nog* promoter, and indeed found significant enrichment of GRHL2 occupation (Fig. 1D).

Previous work in *Drosophila* has shown that GRH remains bound to the promoter of target genes throughout development, with functional transactivation or repression determined through subsequent recruitment of GRH co-factors (Nevil et al., 2017). Although not yet demonstrated for vertebrate GRHL factors, the high conservation of *grh* and GRHL genes suggests this is likely.

Together, our data support a direct relationship between GRHL2 and NOG within the MXP epithelium.

### Disruption of BMP-pSMAD gradients within the maxillary prominence of *Grhl2*^−/−^ embryos

NOG inhibits BMP signalling by sequestering extracellular BMPs, preventing their binding to membrane receptors and phosphorylating SMAD protein effectors. Moreover, epithelial NOG is typically secreted into the mesenchyme to establish BMP gradients and participate in feedback loops, rather than acting locally within the epithelium (He et al., 2010; Matsui and Klingensmith, 2014). Although we did not see a significant difference in total abundance of pSMAD1/5/9 in *Grhl2^−/−^* PA1 (Fig. S2), the spatial distribution of pSMAD5^+^ cells within the MXP of *Grhl2^−/−^* embryos was significantly altered (Fig. 1E,F; Fig. S3). In particular, we noted defined ‘zonal’ expression within the MXP, allowing us to subdivide the MXP into three discrete regions. ‘Zone 1’ includes only the epithelium; ‘Zone 2’ includes neural crest cell (NCC)-derived mesenchyme extending one to five cell diameters from the epithelium; and ‘Zone 3’ includes all remaining MXP mesenchymal cells.

Quantification (Fig. 1G) showed no significant difference in pSMAD5^+^ expression within Zone 1 (*Grhl2^−/−^* 78.2%±0.9%; WT 77.3%±0.7%; mean±s.e.m.). However, *Grhl2^−/−^* embryos presented with significantly more pSMAD5^+^ cells in both Zone 2 (*Grhl2^−/−^* 52.9%±1.6%; WT 32.1%±1.5% *P*<0.01) and Zone 3 (*Grhl2^−/−^* 96.5%±1.9%; WT 73.8%±1.1%; *P*<0.01). These data indicate perturbed SMAD phosphorylation within the MXP of *Grhl2^−/−^* embryos, suggestive of disrupted BMP gradients.

Tightly controlled NOG*/*BMP colocalisation promotes mesenchymal proliferation and survival, and previous studies showed that implantation of NOG-soaked beads into palatal mesenchyme led to increased thickness of overlying epithelium, disruption of BMP signalling (especially BMP4 and BMP7) within the maxillary prominence and concomitant increases in epithelial cell proliferation (Ashique et al., 2002). Moreover, overexpression of *Nog-*encoding retrovirus in limb mesenchyme induces an overgrowth of the apical ectodermal ridge in limb buds (Pizette and Niswander, 1999). These data indicate the existence of reciprocal NOG-mediated feedback loops between mesenchymal and epithelial layers of developing tissues. The role of *Grhl2* in feedback loops within the forming palate is further supported by our previous work showing that the transcription factor ZEB1, expressed exclusively within MXP mesenchyme*,* can restore epithelial fusion in *Grhl2^−/−^* embryos (Carpinelli et al., 2020).

These previous findings support our data that disruption of homeostatic epithelial/mesenchymal BMP signalling in *Grhl2^−/−^* embryos perturbs MXP development.

### *Grhl2*^−/−^*;Nog*^+/−^ mice show rescue of NT and epithelial defects

Given that we saw elevated *Nog* in *Grhl2^−/−^* MXP epithelium, we reasoned that reducing NOG expression would ameliorate *Grhl2^−/−^* epithelial defects. We intercrossed *Grhl2^+/−^;Nog^+/−^* mice, and found that all genotypes were present in approximately Mendelian ratios at E10.5 (Fig. S4; *n*=50 embryos). Strikingly, we found 11 *Grhl2^−/−^;Nog^+/−^* embryos present at E12.5 (*n*=61 embryos), indicative of lethality rescue a full 24 h past the maximum *Grhl2*^−/−^ embryo survival age. These embryos also presented with varying degrees of phenotypic rescue (Table S1). No *Grhl2*^−/−^ embryos (irrespective of *Nog* genotype) were present at E14.5 (*n*=28 embryos).

We hypothesise that increased *Grhl2^−/−^;Nog^+/−^* embryo survival may be related to either improved developmental angiogenesis, or a modicum of heart formation and function rescue. We had previously reported angiogenesis defects in a conditional *Grhl2* lung-deletion model (Kersbergen et al., 2018), and a separate *Grhl2*-deficient mouse model (*Grhl2^1Nisw^*; Pyrgaki et al., 2011) showed substantial defects in heart chamber formation, ventricular wall thickness and development of cardiac vasculature.

**Fig. 2.**
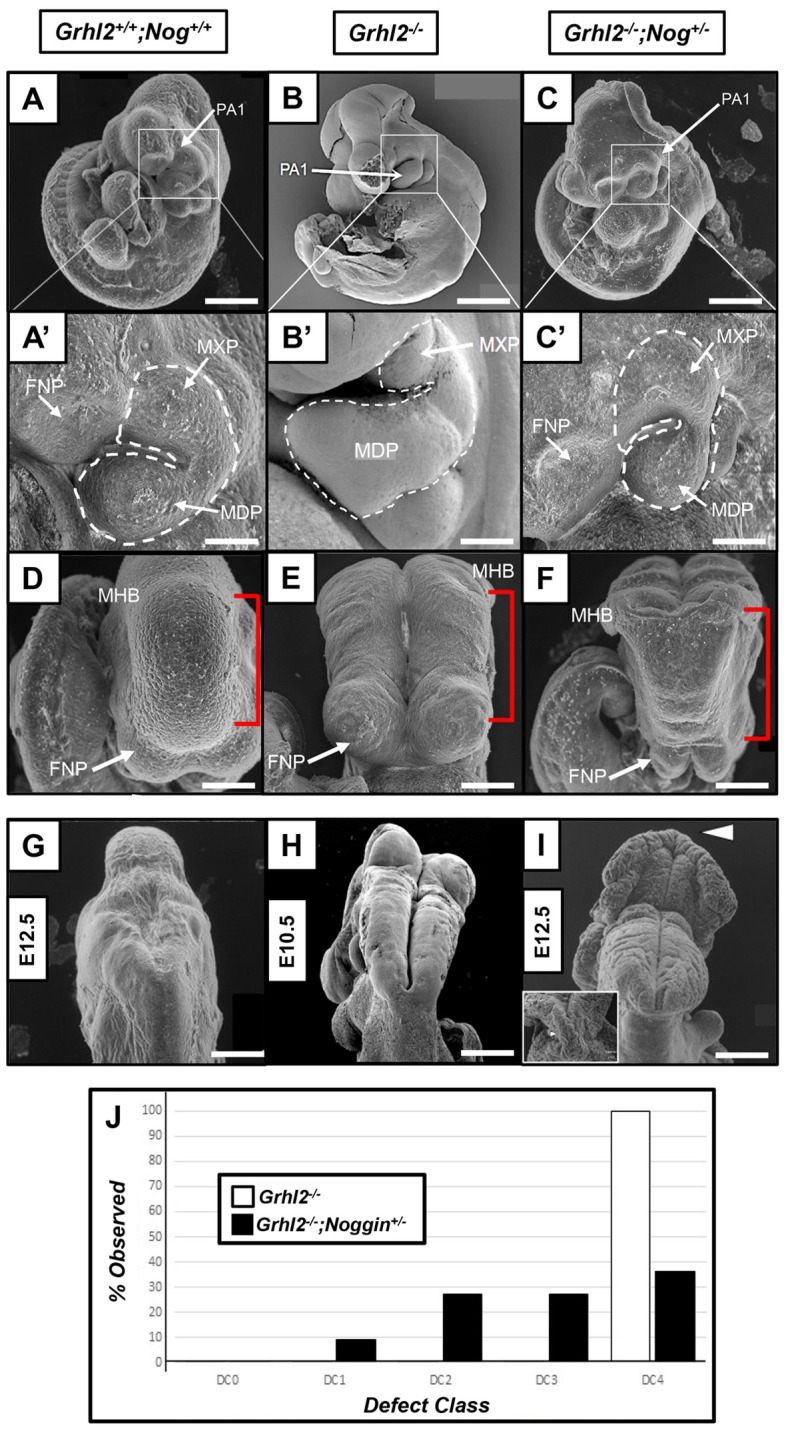
**Scanning electron microscopy shows rescue of *Grhl2^−/−^* phenotypes following reduction of elevated noggin expression.** (A,A′) Control *Grhl2^+/+^;Nog^+/+^* embryo in lateral view (A) shows similarly sized maxillary (MXP) and mandibular (MDP) prominences (A′) at E10.5. (B-C′) In contrast, *Grhl2^−/−^* embryos present with a hypomorphic MXP relative to MDP (B,B′), a phenotype that is rescued following reduction of elevated noggin expression (in *Grhl2^−/−^;Nog^+/−^* embryos; C,C′). (D) Control *Grhl2^+/+^;Nog^+/+^* embryo in dorsal view showing complete cranial NT (red bracket) and fronto-nasal prominence (FNP) fusion. (E) The cranial NT and FNP in *Grhl2^−/−^* embryos remain open (6/6; 100%), although the cranial NT is fused at the approximate level of the future midbrain–hindbrain boundary (MHB). (F) The FNP remained open in the majority of *Grhl2^−/−^;Nog^+/−^* embryos (7/11; 63.6%). (G,H) E12.5 WT embryo in caudal view (G), showing completely fused NT, in contrast with the fully penetrant open NT in *Grhl2^−/−^*embryos (H), which die by E11.5. (I) Rescued *Grhl2^−/−^;Nog^+/−^* embryo at E12.5 presenting with cranial NT fusion at the level of the future MHB (arrowhead). Inset shows dorsal view. (J) Quantitation of phenotypes seen in *Grhl2^−/−^* and rescued *Grhl2^−/−^;Nog^+/−^* embryos by defect severity (defect classes 0-4). DC0: no abnormal phenotype; DC1: cranial dysmorphism (spinal cord, FNP and NT closed); DC2: spinal cord open, FNP and cranial NT closed; DC3: spinal cord open, FNP open, cranial NT closed; DC4: spinal cord open, FNP open, cranial NT open. *Grhl2^−/−^;Nog^+/−^* embryos show evidence of defect rescue (presence of embryos in DC1-3), unlike *Grhl2^−/−^* embryos, which were all classed as DC4. For *Grhl2^−/−^;Nog^+/−^* rescued embryos, *n*=11 embryos (DC0=0, DC1=1, DC2=3, DC3=3, DC4=4). Scale bars: 1 mm (A-C,G-I); 250 μm (A′-C′); 500 μm (D-F).

*Grhl2^−/−^;Nog^+/−^* embryos typically showed substantial normalisation of maxillary hypomorphism (Fig. 2A-F). Moreover, we also observed 7/11 *Grhl2^−/−^;Nog^+/−^* embryos with variable fusion of the frontonasal process (FNP), cranial NT and spinal cord, or combinations of these (Fig. 2G-I), allowing us to characterise rescued phenotypes into one of five ‘defect classes’ (DCs; Table S1 and Fig. S5): DC0 (no abnormal phenotype), DC1 (cranial dysmorphism – spinal cord, FNP and NT closed), DC2 (spinal cord open, FNP and cranial NT closed), DC3 (spinal cord open, FNP open, cranial NT closed) and DC4 (spinal cord open, FNP open, cranial NT open).

All *Grhl2^−/−^;Nog^+/+^* embryos presented as DC4, indicative of no rescue. Conversely, only 4/11 (36.4%) *Grhl2^−/−^;Nog^+/−^* embryos presented as DC4, with 7/11 displaying variable rescue: cranial NT fusion (DC3; 3/11); FNP and cranial NT fusion (DC2; 3/11); and full FNP, cranial and spinal fusion (DC1; 1/11, 9%; Table S1). *Grhl2^−/−^;Nog^−/−^* embryos did not present with substantial rescue of *Grhl2*-dependent phenotypes in any of the FNP, cranial NT or spinal cord (4/5, 80%, as DC4). *Nog*-null embryos present with defects of cranial NT fusion, manifesting as exencephaly (McMahon et al., 1998; Tylzanowski et al., 2006). Loss of *Nog* exclusively within the neural crest also results in craniofacial defects, including clefting (Graf et al., 2016; He et al., 2010; Matsui and Klingensmith, 2014; McMahon et al., 1998), suggesting that one reason for lack of rescue in *Grhl2^−/−^;Nog^−/−^* embryos may be the presence of additive mesenchyme-specific defects as a consequence of full *Nog* loss, irrespective of *Grhl2* involvement. Additionally, given that overexpression of *Nog* in palatal mesenchyme expression also leads to tissue fusion defects (Li et al., 2021), it is clear that tightly regulated homeostatic expression of *Nog* is essential for correct tissue fusion in development. Therefore, our data suggest that at least one allele of *Nog* (50% gene dosage) is required to establish, or maintain, homeostatic BMP-pSMAD gradients within both the developing neuroectoderm and palatal primordia in *Grhl2^−/−^* embryos.

We observed rescue of MXP/mandibular prominence (MDP) epithelial morphology and increased NT folding in *Grhl2^−/−^;Nog^+/−^* embryos (Fig. S6). Previous work has shown that NOG-mediated BMP modulation is essential for dorsolateral hinge point formation and bending in cranial NT fusion (Stottmann et al., 2006; Ybot-Gonzalez et al., 2007). This may be the mechanism by which *Grhl2^−/−^;Nog^+/−^* cranial NT fusion is rescued. Indeed, within dorsal NT closure, BMP signalling actually promotes *Nog* expression, suggesting that epithelial regulatory feedback mechanisms include BMP signalling itself (Stottmann et al., 2001). Likewise, in the palate, NOG/BMP regulation differentially modulates cellular proliferation/apoptosis between the anterior and posterior regions of the palatal shelves (He et al., 2010).

Taken together, our data indicate that reducing elevated NOG expression in *Grhl2^−/−^* embryos ameliorates craniofacial and NT fusion defect severity, and identify a previously unappreciated genetic interaction driving epithelial tissue fusion.

### *Grhl2*^−/−^ *ex vivo* maxillary culture recapitulates *in vivo* epithelial defects

**Fig. 3.**
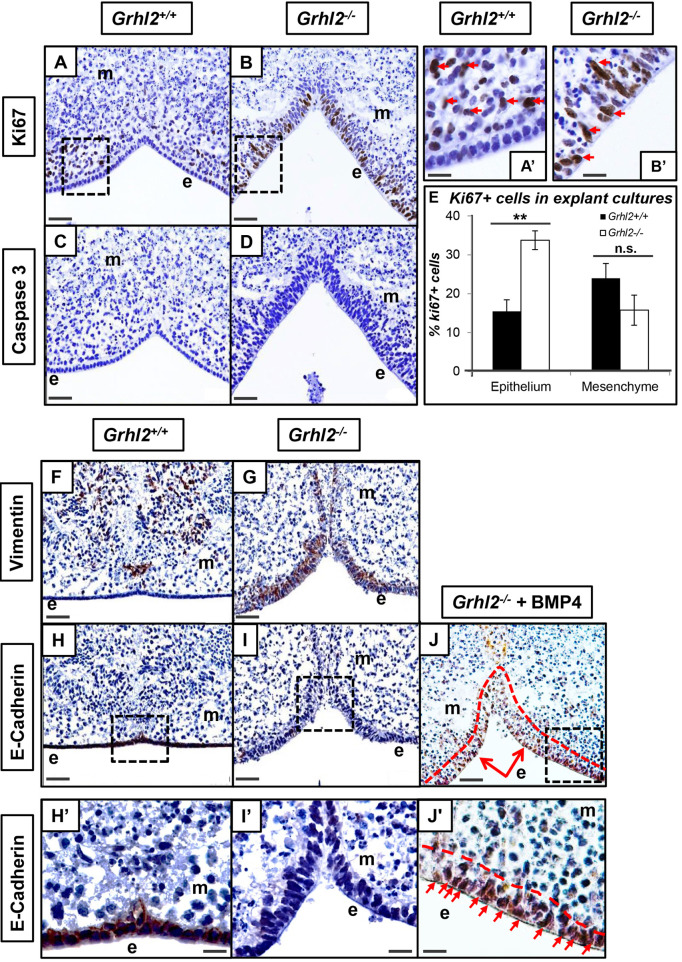
**Cultured *ex vivo* tissue explants recapitulate the *in vivo Grhl2^−/−^* epithelial defect.** (A,B) Cellular proliferation (Ki67^+^ cells) is confined predominantly to the mesenchymal cell layer adjacent to the epithelium, but not within the epithelium itself (A,A′; arrows) in WT embryos. In contrast, the ‘epithelium’ of *Grhl2^−/−^* embryos shows a large number of proliferating cells (B,B′; arrows). Boxed regions of in A,B are magnified in A′,B′. (C,D) No apoptosis (caspase 3*^+^* cells) is observed within explant tissue from either WT or *Grhl2^−/−^* embryos. (E) Quantitation of Ki67^+^ proliferating cells in explant cultures. Error bars indicate s.d. **P≤0.01, (unpaired, two-tailed Student's *t*-test). n.s., not significant. (F,G) As is seen *in vivo*, the mesenchymal marker vimentin is expressed within the neural-crest derived mesenchyme of PA1 in WT embryos (F). However, vimentin is expressed throughout the outermost ‘epithelial’ layer in *Grhl2^−/−^* embryos (G). (H,I) E-cadherin is expressed throughout the epithelial layer of WT (H) but not *Grhl2^−/−^* (I) embryos. (J) However, supplementation of *Grhl2^−/−^* embryo explant media with soluble BMP4 led to re-expression of E-CAD within the outermost ‘epithelial’ cell layer (red dashed line; arrows). (H′-J′) Higher magnification views of the boxed regions in H-J. e, epithelial cell layer; m, mesenchymal cells. All sections and immunohistochemistry are representative of a minimum of *n*=3 biological replicates per genotype. Scale bars: 40 μm (A-J); 15 μm (A′-B′,H′-J′).

To circumvent the E11.5 embryonic lethality of *Grhl2*^−/−^ embryos, we adapted existing *ex vivo* palate explant models to examine pre-palatal primordia from E10.5 embryos (de Vries and Dworkin, 2022) (Fig. S7). The thickening of the outermost MXP layer in *Grhl2^−/−^* embryos *in vivo* is most likely due to increased proliferation, consistent with the known role of *Grhl2* as a proliferation suppressor (Xiang et al., 2012). We have shown previously that the cells in the ‘epithelial’ layer in *Grhl2^−/−^* embryos are present, and retain an intact basal lamina (Carpinelli et al., 2020), allowing us to demarcate accurately the separation between epithelial and mesenchymal cells (Carpinelli et al., 2020). Therefore, we examined markers of proliferation (Ki67, also known as Mki67; Fig. 3A-B′) and apoptosis (activated caspase 3; Fig. 3C,D) in MXP explants. We found significantly increased proliferation in the epithelial layer (‘Zone 1’) of *Grhl2^−/−^* MXP (33.75%±2.4%; mean±s.e.m.) relative to the epithelial layer of *Grhl2^+/+^* MXP (14.5+2.94%; *n*=3; *P*<0.05; Fig. 3E). Conversely, the proportion of proliferating cells within the mesenchymal cells adjacent to the epithelium (‘Zone 2’) showed a non-significant decrease in *Grhl2^−/−^* MXP (15.8%±3.9%) relative to *Grhl2^+/+^* MXP (23.9%±3.8%; *n*=3; *P*>0.05), strongly supporting our hypothesis. No apoptosis was detected in either WT or *Grhl2^−/−^* explants (Fig. 3C,D).

*Grhl2^−/−^* MXP epithelium shows morphology and cell-identity defects, gain of mesenchymal vimentin (VIM) expression and loss of epithelial E-cadherin (E-CAD; CDH1) expression (Carpinelli et al., 2020). We analysed cellular morphology and VIM/E-CAD expression in our *Grhl2^−/−^* explants and found that these neatly phenocopied *in vivo Grhl2^−/−^* MXP development (Fig. 3F-I′). We investigated whether modulating the NOG-BMP pathway could overcome *Grhl2* loss, and treated *Grhl2^−/−^* explants with recombinant BMP4 (Fig. 3J,J′). After 4 days culture, we found substantial rescue of E-CAD expression throughout the ‘epithelial’ layer (Fig. 3J,J′), indicating that BMP supplementation may partially restore epithelial identity in *Grhl2^−/−^* explants.

**Fig. 4.**
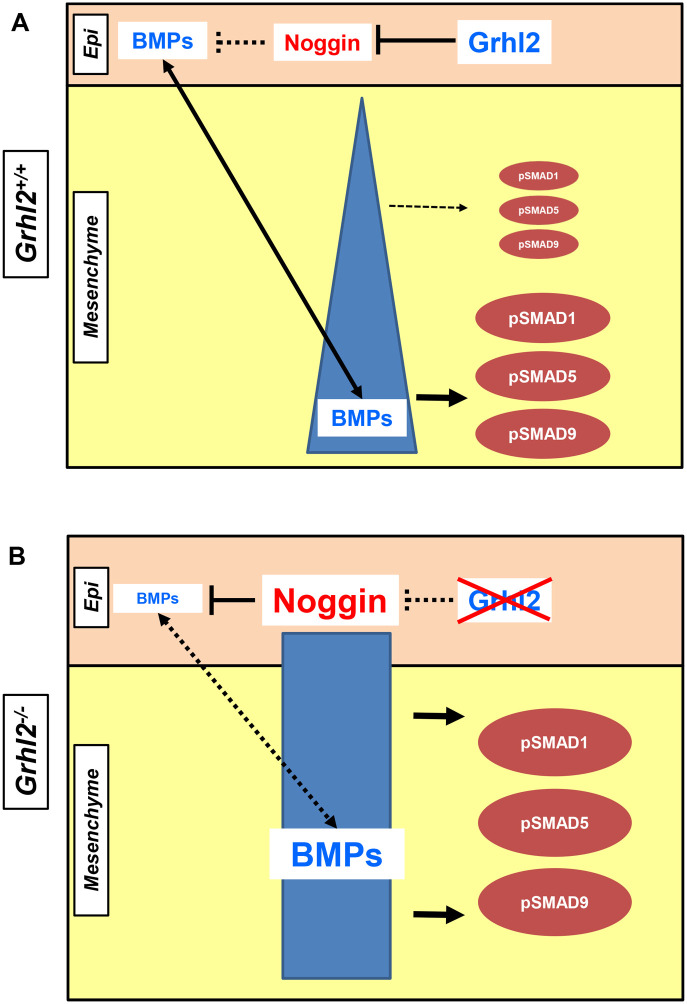
**Hypothesised GRHL2-NOG interaction in palatal development.** (A) In *Grhl2^+/+^* palatal epithelium (Epi), GRHL2 represses NOG expression, leading to increased epithelial BMP signalling. This allows for maintenance of a homeostatic feedback loop that establishes and maintains a BMP gradient (blue triangle) and subsequent differences in SMAD phosphorylation between the epithelium and mesenchyme. (B) In *Grhl2^−/−^* embryos, GRHL2 signalling is disrupted, leading to weakening or compromise of the periderm. Moreover, GRHL2 loss results in NOG de-repression, leading to NOG overexpression, decreased epithelial BMP signalling to the mesenchyme, abolition of the BMP feedback loop and disruption of the BMP gradient (blue column), resulting in aberrantly localised SMAD phosphorylation throughout the pre-palatal mesenchyme.

Our model (Fig. 4) suggests that GRHL2 represses NOG in palatal epithelium, allowing epithelial BMP to drive a BMP gradient within the mesenchyme, leading to regional activation of pSMAD (Fig. 4A). This hypothesis is supported by the fact that *Grhl2* is expressed only in the epithelium, and tissue-specific inactivation of *Grhl2* within NCCs does not lead to defects (Carpinelli et al., 2020). Loss of GRHL2 leads to premature de-repression of epithelial NOG and reduced epithelial BMP signalling, leading to disruption of tight homeostatic epithelial/mesenchymal BMP feedback loops (Fig. 4B). This leads us to hypothesise that cells within the outermost MXP layer may have been initially established as ‘epithelial’ cells, and have undergone epithelial-to-mesenchymal transition during embryogenesis (as a result of a lack of *Grhl2*) to take on more mesenchymal characteristics, thereby presenting with phenotypes intermediate between those of WT epithelial and mesenchymal cells.

Although clearly a reductionist model (as many other target genes influence cell fate downstream of epithelial GRHL2 signalling), it nonetheless proposes how the GRHL2/NOG/BMP axis may operate within the pre-palatal craniofacial primordia. Other noggin pathway genes, such as activin B genes, *Nodal* and *Wnt8* (Bayramov et al., 2011), or factors regulated by BMPs, such as *Satb2*, *Smad6*, *Hand1*, *Gadd45g* and *Gata3* (Bonilla-Claudio et al., 2012), could also operate within this pathway. Moreover, previous studies have also showed that BMP signalling drives ΔNp63 expression in zebrafish (Bakkers et al., 2002), and *Grhl2* and *p63* (*Trp63*) share complex reciprocal feedback (Mehrazarin et al., 2015). Therefore, aberrant p63 expression may further underpin abnormal *Grhl2^−/−^* epithelia.

Our current and future directions will involve culturing and analysing explants from *Grhl2^−/−^;Nog^+/−^* mice to further delineate some of these signalling pathways, as well as supplementing explants with BMP2/4/7 (singly and in concert) to determine phenotypic rescue, E*-*CAD*/*VIM expression, cellular morphology and ability to fuse, in order to determine the cellular and molecular effects reduction of elevated NOG expression may have on *Grhl2*-dependent palatal development.

## MATERIALS AND METHODS

### Animals

Animal colonies were maintained in accordance with government regulations at Monash (E/1585/2015/M) and La Trobe (AEC 16-72 and AEC 16-19) Universities. Mice used were *Grhl2^tm1.1Jane^* (Rifat et al., 2010) and *Nog^tm1Amc^* (McMahon et al., 1998), maintained as *Grhl2^+/−^;Nog^+/−^* adults (of both sexes) on a C57/Bl6 background at La Trobe University for >15 generations. Double-heterozygous adults were viable and fertile, and presented with no visible phenotypes.

### Antibodies

Primary antibodies used within this study were: anti-E-cadherin [rabbit monoclonal antibody (mAb), #3195, Cell Signaling Technology], anti-vimentin (rabbit mAb, #5741, Cell Signaling Technology), anti-cleaved caspase 3 (rabbit mAb, #9664, Cell Signaling Technology), anti-Ki67 (rabbit mAb, #9729, Cell Signaling Technology), anti-pSmad1/5/9 (rabbit mAb, #9511S, Cell Signaling Technology), anti-pSMAD5 (rabbit mAb, ab92698, Abcam), anti-Sox9 (rabbit polyAb, ab26414, Abcam) and anti-β-actin (rabbit mAb HRP conjugate, #5125S, Cell Signaling Technologies). Secondary antibodies used were: goat anti-rabbit biotinylated IgG (BA-1000, Vector Laboratories), goat anti-rabbit HRP-conjugated IgG (ab97051, Abcam) and Alexa Fluor 488 goat anti-rabbit IgG (ab150077, Abcam).

### Chromatin immunoprecipitation (ChIP)

Dissected E8.5 first pharyngeal arch tissue (including paired maxillary processes and mandibular process) was pooled from eight embryos. Tissue was homogenised and cross-linked in 1 ml 1% formaldehyde in PBS for 15 min at room temperature (RT). All following steps were also conducted at RT unless otherwise specified. Fixation was quenched by addition of 106 µl of 1.375 M glycine for 5 min. The sample was centrifuged at 800 ***g*** for 5 min and the resultant pellet washed twice with ice-cold PBS supplemented with protease inhibitors (cOmplete Protease inhibitor cocktail, Roche). The tissue pellet was resuspended in 200 µl 1% SDS lysis buffer (1% SDS, 10 mM EDTA, 50 mM Tris-HCl pH 8.1, 1×protease inhibitor, 10 mM sodium butyrate) and incubated on ice for 10 min before DNA shearing by sonication. Sonication was performed at 4°C and on ice using a Branson 250 sonicator equipped with a double stepped microtip (3 mm tip diameter) at 10% power and 90% duty cycle (2×11 pulses, repeated a total of five times with resting on ice for 2 min between each exposure). This was followed by centrifugation at 16,000 ***g*** for 10 min at 4°C. Next, the sheared and lysed protein-DNA fragments were removed with the supernatant and made up to a total volume of 2 ml with ChIP dilution buffer (0.01% SDS, 1.1% Triton X-100,1.2 mM EDTA, 16.7 mM Tris-HCl pH 8.1, 167 mM NaCl,1×cOmplete Protease inhibitor cocktail, 10 mM sodium butyrate). A 100 µl aliquot was taken to serve as the ‘input’ for later analysis and was stored at −20°C until required. To minimise non-specific binding the sample was incubated with protein A-magnetic beads for 2 h at 4°C with constant gentle agitation. The magnetic beads were removed and the sample was separated into two aliquots, which were incubated with either anti-Grhl2 (#HPA004820, Sigma-Aldrich, 1:40) or non-specific IgG (#3900S, Cell Signaling Technology, 1:200) antibodies for a minimum of 16 h at 4°C with constant gentle agitation. Next, samples were incubated with fresh protein A-magnetic beads for 2 h at 4°C with constant gentle agitation. The magnetic beads were washed in sequence with low-salt wash buffer (0.1% SDS, 1% Triton X-100, 2 mM EDTA, 20 mM Tris-HCl pH 8.1, 150 mM NaCl), high-salt wash buffer (0.1% SDS, 1% Triton X-100, 2 mM EDTA, 20 mM Tris-HCl pH 8.1, 500 mM NaCl), lithium chloride wash buffer [0.25 M LiCl, 1% (v/v) IGEPAL, 10 mM Tris-HCl pH 8.1, 1 mM EDTA, 1% (w/v) deoxycholic acid] and Tris-EDTA (TE) buffer (10 mM Tris; 1 mM EDTA), each for 5 min. Immune complexes were isolated using two washes of 100 µl of elution buffer (1% SDS, 0.1 M NaHCO_3_) at 65°C for 10 min with constant agitation. Sample and input aliquots were then treated with NaCl (0.4 M final concentration, 65°C for 4 h with constant agitation) to reverse the cross-linking before treatment with RNase A (100 µg/ml for 30 min at 37°C with constant agitation) and proteinase K (100 µg/ml 30 min at 45°C with constant agitation). DNA fragments were isolated using 222 µl phenol/chloroform/isoamyl alcohol (25:24:1, pH 8, Sigma-Aldrich) and precipitated with 1/10 volume 3 M sodium acetate (pH 5.2) and 2.2 volumes 100% ethanol, supplemented with 1 µl glycogen and pelleted at 16,000 ***g*** for 15 min. DNA pellets were washed with 70% ethanol and allowed to air dry for 10 min before resuspension in 50 µl 10 mM Tris (pH 7.4).

Quantitative Real-Time PCR (Q-RT-PCR) was performed to assess the relative enrichment of the *Nog* locus at the predicted binding site (AACTAGTT; chr11:89,289,343-89,289,336; 11,295 kb upstream of the TSS) in the anti-Grhl2 treated and non-specific IgG antibody-treated aliquots versus the input sample, using *Arhgef19* (a known GRHL­ gene target; Boglev et al., 2011; Caddy et al., 2010) as a positive control and *MyoD* (*Myod1*) as a negative control for GRHL2 binding. Each reaction was performed in triplicate on a CFX96 Real-Time system with a C1000 thermocycler (Bio-Rad). Analysis was performed as described previously (Dworkin et al., 2007).

### Western blotting

Protein lysate from whole first pharyngeal arch of both *Grhl2*^+/+^ and *Grhl2*^−/−^ embryos was extracted by first homogenising the tissue by repeated pipetting followed by two freeze-thaw cycles, freezing at −80°C and allowing to thaw slowly on ice. The tissue lysate from three embryos were pooled to ensure sufficient protein concentration. Pooled samples were lysed using RIPA lysis buffer supplemented with protease and phosphatase inhibitor cocktails (cOmplete Protease inhibitor cocktail, Roche, and PhosSTOP, Roche, respectively).

Protein concentration was estimated using the Bio-Rad DC colorimetric protein assay (5000112, Bio-Rad), which is a modified version of the Lowry protein assay (Lowry et al., 1951). Assay protein standards were made from serial dilutions of 2 mg/ml bovine serum albumin (BSA) in H_2_O to generate 0.4 mg/ml, 0.8 mg/ml, 1.2 mg/ml, 1.6 mg/ml and 2 mg/ml standard concentrations. Protein lysate was diluted 1:10 in H_2_O and 5 µl was pipetted in triplicate into a 96-well, clear, flat-bottom plate, along with the known-concentration BSA samples. The reagent solutions were added to each well, as per manufacturer's specifications, and allowed to develop at room temperature for 15 min before spectrophotometric absorbance readings were taken at 750 nm wavelength using a Fluostar Omega plate reader (BMG Labtech). Sample protein concentration was estimated by preparing a standard curve using the known-concentration BSA samples and averaging triplicate readings. The pooled sample lysates were estimated at ∼2.4 µg/µl (WT: 2.38 µg/µl; knockout: 2.37 µg/µl) and 20 µl was used when loading gels (NuPAGE Bis Tris 10% pre-cast gels, Invitrogen), totalling approximately 48 ng protein per well.

The gel was immersed in 1×NuPAGE running buffer (diluted from 20× stock: 52.3 g/250 ml MOPS, 30.3 g/250 ml Tris base, 5 g/250 ml SDS, 1.5 g/250 ml EDTA) and the wells were loaded with PageRuler pre-stained protein ladder (10-250 kDa, Thermo Fisher Scientific) and the pooled protein lysate. The gels were run at 160 V using a PowerPac Basic power supply (Bio-Rad) for 1 h or until the PageRuler dye reached the bottom of the gel. Next, the protein was transferred to methanol activated polyvinylidene difluoride (PVDF) membrane (Bio-Rad) using 100 V at 4°C for 1.5 h on the PowerPac Basic power supply (Bio-Rad) in 1×transfer buffer (diluted from 20× stock: 0.2 g/125 ml bicine, 13.1 g/125 ml Bis-Tris, 0.75 g/125 ml EDTA), with constant gentle stirring. Membranes were blocked with 5% (w/v) BSA and washed with PBS (two 5 min washes with agitation) before incubating overnight at 4°C with the primary antibody in Tris-Buffered Saline supplemented with 10% Tween-20 (TBST). Two 5 min washes with PBS were performed before incubating the membrane with HRP-conjugated anti-rabbit secondary for 1 h at room temperature. The membrane was washed again (two 5 min washes in PBS) before treating with enhanced luminol-based chemiluminescent (ECL) Western Blotting Substrate Kit (Abcam) for 5 min and imaged using the Bio-Rad Chemidoc XRS+ system with both colorimetric (ladder bands) and chemiluminescence (target bands) outputs.

The membrane was washed with PBS (5 min, twice), methanol (5 min) and PBS again (5 min, twice) before blocking with 5% BSA to prepare for re-probing with loading control primary antibody (β-actin; 1:10,000) as a loading control.

The images were processed and further analysed using Image Lab software (Bio-Rad). Densitometric analysis was conducted using Image Lab software following the manufacturer's recommendations as well as a previously described procedure (Taylor et al., 2013).

### Immunohistochemistry

All steps were conducted at room temperature unless otherwise stated, and de-waxing and rehydration were performed using standard methods. For antigen retrieval, slides were immersed in 250 ml citrate antigen retrieval buffer [18 mM citric acid, 8.2 mM sodium citrate, 0.5% (v/v) Tween 20] and heated using a Panasonic 1100 W microwave at 90% power for 3 min and 20 s to reach boiling and then reduced to a light boil at 30% power for 5 min to expose antigenic epitopes. Slides were cooled for 20 min at RT and washed three times, 5 min each wash, in PBS, and then immersed in 3% (v/v) H_2_O_2_ in PBS for 10 min to quench endogenous peroxidase activity, and two 5 min washes in PBS. Non-specific antibody binding was minimised using a blocking solution comprising diluted normal goat serum (5% in PBS) for 30 min. The slides were incubated with the primary antibody in the recommended blocking solution concentration for a minimum of 1 h at 4°C. Slides were washed with PBS (two 5 min washes) before incubation with a biotin-conjugated secondary antibody, diluted in the same blocking solution concentration as the primary incubation step, for 30 min. The slides were washed three times, 5 min each wash, in PBS, incubated with avidin/biotinylated enzyme complex (ABC; Vector Labotaries) solution for 30 min and washed again three times, 5 min each wash, in PBS. The slides were then immersed in diaminobenzidine (DAB; Vector Laboratories) substrate for 2 min, washed twice, 2 min each wash, in H_2_O, counterstained with Haematoxylin for 3 s, rinsed for 30 s in H_2_O, blued in 1× Scott's Blue Substitute (Sigma-Aldrich) for ∼10 s, before final washing three times, 2 min each wash, in H_2_O. Slides were then dehydrated in 100% ethanol (3×2 min) and cleared in xylene (3×2 min) to allow for long-term coverslip mounting in ‘DPX new’ non-aqueous mounting medium (Sigma-Aldrich).

### Maxillary explant culture

These were performed as described previously (de Vries and Dworkin, 2022). Briefly, litters were collected at E10.5 and the palate tissue dissected. Using precision needles and fine-tipped tweezers, a slice containing the maxillary and nasal processes was dissected from *Grhl2^+/+^* and *Grhl2^−/−^* E10.5 embryos, with care taken to retain as little mandibular and dorsal NT tissue as possible.

Tissue explants were transferred to a 6-well tissue culture plate (clear, flat-bottom, Corning Costar) with 1 ml Dulbecco's Modified Eagle Medium (DMEM, Gibco) containing 6 mg/ml BSA, 1% (v/v) ITS-G (41400-045, Gibco), 140 μg/ml ascorbic acid (A92902, Sigma-Aldrich) and 100 units/ml penicillin/streptomycin (15140-122, Gibco). Explants were incubated at 37°C in 5% CO_2_ for up to 5 days, with media changed daily. For supplementation with bone morphogenetic protein, recombinant mouse BMP4 (SRP3298, Sigma-Aldrich) was added to the culture media to a final concentration of 5 ng/ml and replenished daily with the media changes. Palate explants were fixed in 4% (w/v) paraformaldehyde at 4°C overnight then orientated in 2% (w/v) low melting point agarose in PBS before paraffin processing utilising standard methods.

### Microscopic imaging

Whole embryo or *ex vivo* tissue samples were imaged using a Nikon SMZ18 stereoscopic microscope equipped with a Nikon DS-Ri2 camera. Tissue sections were imaged using a Nikon Ti Eclipse equipped with a Nikon DS-Qi1Mc monochrome camera for fluorescence imaging and a Nikon DS-Fi1 high resolution colour camera for imaging of stained samples. Images were collected using Nikon NIS-Elements image software.

### Scanning electron microscopy

All characterisation and analysis of craniofacial phenotypes was performed by an operator unaware of genotype, as embryos were classified based on phenotypic presentation immediately during harvest (prior to genotyping). Embryos were fixed overnight in 2.5% (w/v) glutaraldehyde in PBS at 4°C and were subsequently rinsed twice in PBS for at least 10 min each. Embryos were dehydrated in the following washes: 50% ethanol (30 min), 70% ethanol (30 min), 95% ethanol (30 min) and 100% ethanol (2×30 min). Embryos were then incubated in a 1:1 100% ethanol:hexamethyldisilazane (HMDS; Pro Sci Tech) solution for 15 min, followed by two washes in 100% HMDS for 15 min. Excess HMDS was removed and the embryos were left to dehydrate in a fume hood overnight. Dehydrated embryos were mounted onto stubs with carbon tabs and visualised using a Hitachi TM3030Plus Tabletop scanning electron microscope, detecting secondary emission (SE) at 15 kV, at the La Trobe Institute of Molecular Science (LIMS) Bioimaging facility.

### Immunohistochemistry quantitation

All cell counts for both pSMAD5^+^ and Ki67 quantitation were performed manually. For pSMAD5, both pSMAD5^+^ and pSMAD5^−^ cells within each zone outlined in Fig. 1E,F were counted (within all MXP tissue visible in each section), from *n*=3 biological replicates per genotype. For Ki67 quantitation, three independent fields were selected per section (slightly larger than the boxed regions shown in Fig. 3A,B), comprising both the epithelial region and adjoining mesenchyme, and all Ki67^+^ and Ki67^−^ cells were counted. These counts were also performed from *n*=3 biological replicates per genotype.

### Statistical analysis

Chi-squared tests (*χ*^2^) were used to analyse the genotypic ratios of experimental crosses. A threshold of *P*<0.05 was used and test statistics returned below this indicated the genotypic frequency of the cross was not consistent with Mendelian inheritance. Quantitative data was derived from at least three independent experiments. Descriptive statistics are mean±s.e.m. of data for (*n*) individuals or (*n*) independent experiments, unless otherwise specified. Microsoft Excel was used for unpaired, two-tailed Student's *t*-tests, with normally distributed continuous variables. *P*-values are given as follows: *P≤0.05, **P≤0.01, ***P≤0.001.

## Supplementary Material



10.1242/develop.202420_sup1 Supplementary information
