# Supplementary material for: Grainyhead-like 2 interacts with noggin to regulate tissue fusion in mouse
Source: Development. 2024 Feb 28;151(5):dev202420. doi: 10.1242/dev.202420 (PMC10946436; doi:10.1242/dev.202420)
Supplement: Supplementary information [file develop-151-202420-s1.pdf]

A

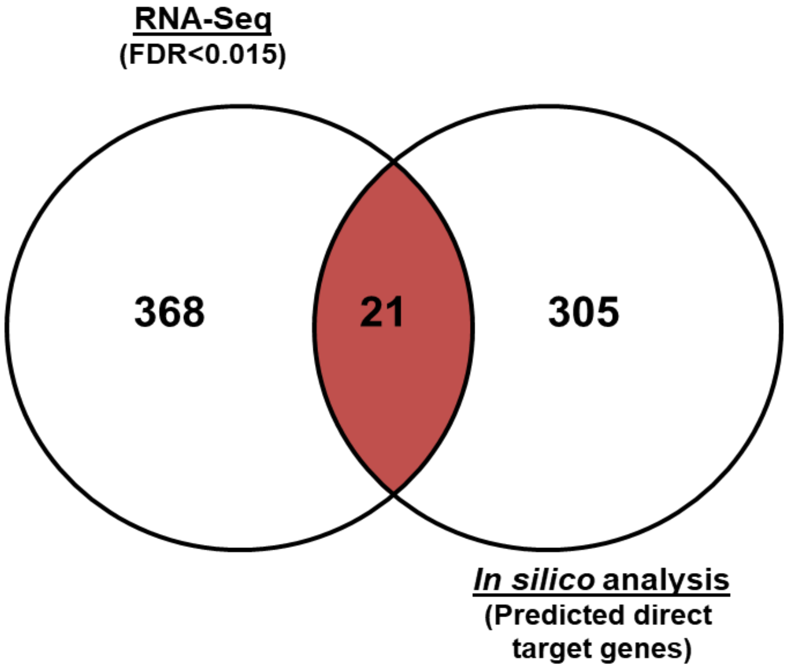

B

Gene Symbol	Gene Name	Fold-change
Ovol1	*OVO homolog-like 1 (Drosophila)*	-3.760
Arhgef19	*Rho guanine nucleotide exchange factor*	-3.753
Cldn4	*Claudin 4*	-3.326
Pvrl4	*Poliovirus receptor-related 4*	-3.126
Grhl2	*Grainyhead-like 2*	-3.027
Ppl	*Periplakin*	-2.817
Cdh1	*E-Cadherin*	-2.471
Tbc1d2	*TBC1 domain family, member 2*	-1.138
Tfap2c	*Transcription factor AP-2, gamma*	-0.996
Sh3bgrl2	*SH3 domain binding glutamic acid-rich protein like 2*	-0.473
Nhs	*Nance-Horan Syndrome protein*	-0.336
Rbm18	*RNA binding motif protein 18*	-0.210
Rcc2	*Regulator of chromosome condensation 2*	-0.186
Nr2f2	*Nuclear receptor sub-family 2, group F, member 2*	0.308
Mxra8	*Matrix-remodelling associated protein 8*	0.349
Vat1	*Vesicle amine transport protein 1 homolog*	0.364
Kctd12b	*Potassium channel tetramerisation domain containing protein 12b*	0.384
Npr3	*Natriuretic peptide receptor 3*	0.652
Cbln1	*Cerebellin 1 precursor proteim*	1.019
Nog	***Noggin***	**1.318**
Klhdc8a	*Kelch domain containing protein 8a*	3.000

Fig. S1. RNA-seq data paired with *in silico* predictive techniques identifies *Noggin* as a putative direct target of *Grhl2* in the developing first pharyngeal arch. (A) RNA-seq data set of *Grhl2*$^{-/-}$ first pharyngeal arch (PA1) tissue identified 368 differentially-regulated genes, relative to PA1 extracted from WT littermates (n=5 per genotype). When this dataset was cross-referenced against a list of 305 putative target genes (those that contain a conserved *Grhl*-binding site within their promoters), 21 genes were present on both lists. (B) The 21 genes that were differentially regulated in PA1 tissue of *Grhl2*$^{-/-}$ embryos. *Nog* is the second-most highly upregulated gene.

Development • Supplementary information

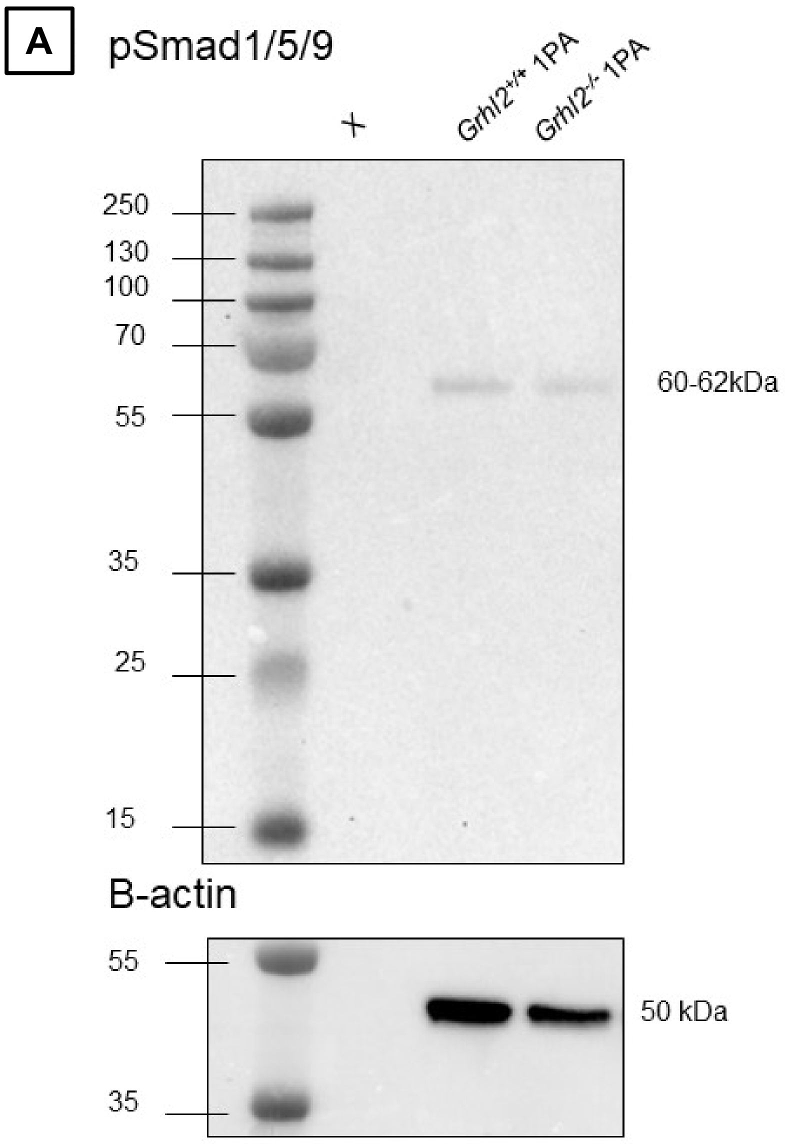

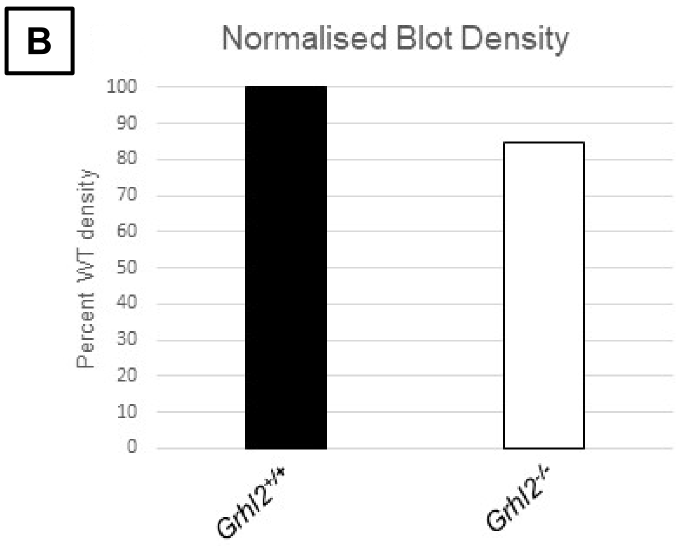

Fig. S2. Western Blot and densitometric scanning of pSMAD1/5/9 in *Grhl2*$^{+/+}$and *Grhl2*$^{-/-}$pharyngeal arches. (A-B) pSMAD1/5/9 (~62kDa) expression in lysate pooled from n=5 arches relative to β-actin. pSmad1/5/9 is not significantly decreased in E10.5 *Grhl2*$^{-/-}$ PA1 tissue compared to WT PA1, relative to β-actin (50kDa) by densitometric quantitation of normalised Western Blot density.

Development • Supplementary information

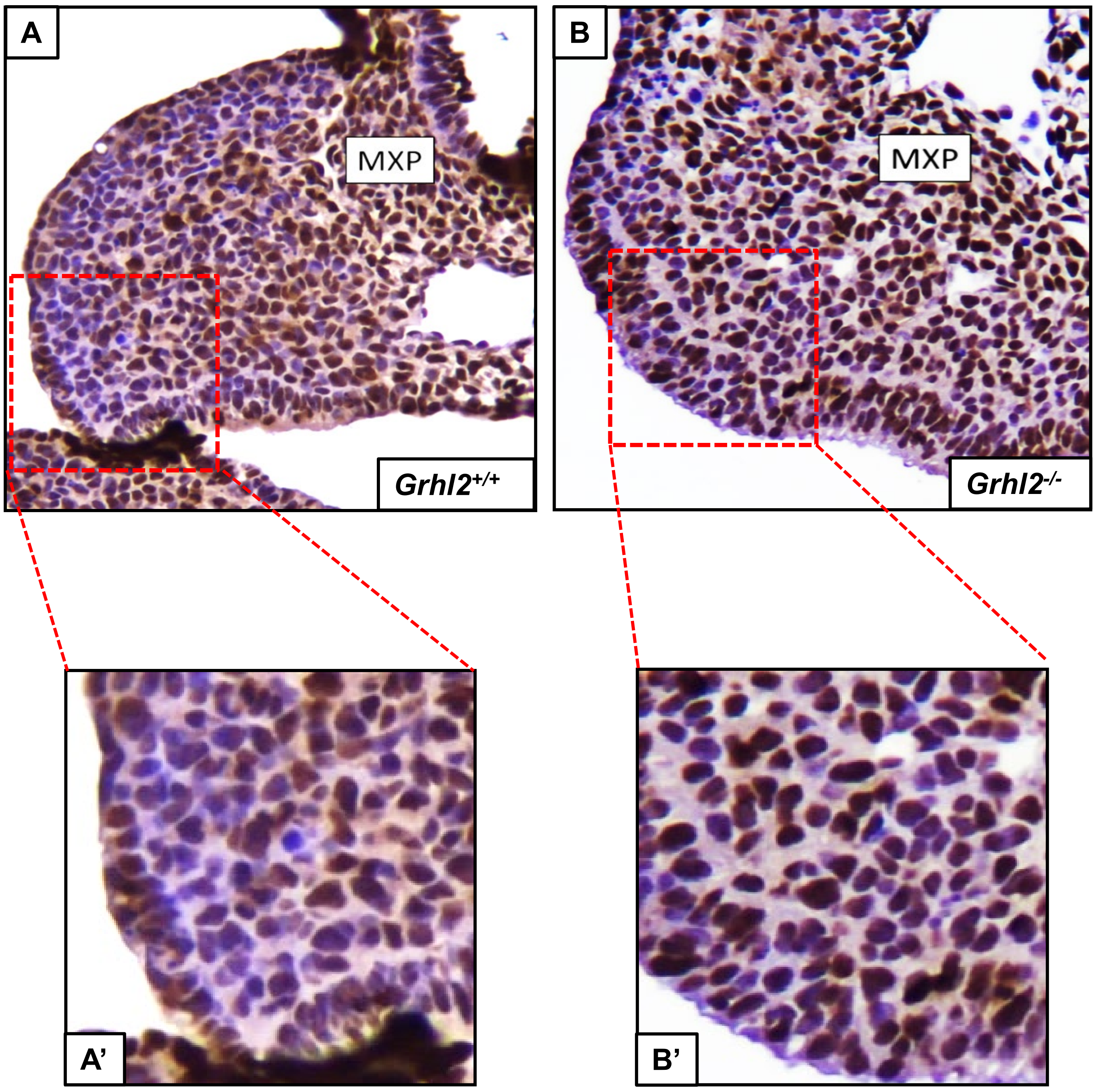

Fig. S3. pSmad5 expression in the maxillary prominence (MXP) of *Grhl2*$^{+/+}$and *Grhl2*$^{-/-}$ tissue (A-B) Non-demarcated images of pSMAD5 expression in *Grhl2*$^{+/+}$(A) and *Grhl2*$^{-/-}$ (B) MXP. This image is a non-annotated version of Fig. 1F-G from the main paper. Magnified images of boxed regions shown in A-B.

Development • Supplementary information

Aa)

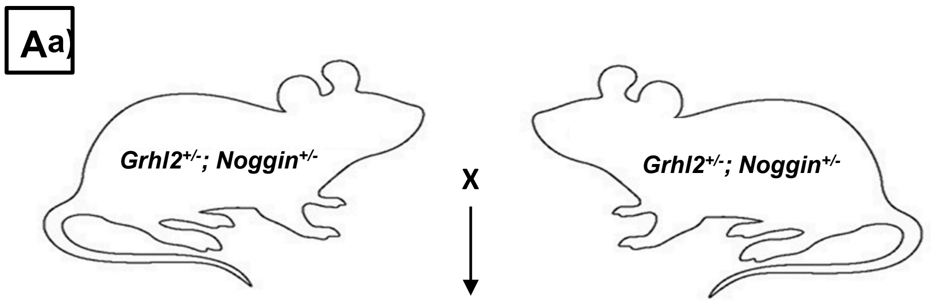

B

E10.5

Genotype	Obs	Exp	Obs%	Exp%	χ^2
***Grhl2*$^{+/+}$*; Nog*$^{+/+}$**	2	3.125	4.00%	6.25%	0.4492
***Grhl2*$^{+/+}$*; Nog*$^{+/-}$**	8	6.25	16.00%	12.5%	
***Grhl2*$^{+/+}$*; Nog*$^{-/-}$**	1	3.125	2.00%	6.25%	
***Grhl2*$^{+/-}$*; Nog*$^{+/+}$**	6	6.25	12.00%	12.5%	
***Grhl2*$^{+/-}$*; Nog*$^{+/-}$**	13	12.5	26.00%	25%	
***Grhl2*$^{+/-}$*; Nog*$^{-/-}$**	3	6.25	6.00%	12.5%	
***Grhl2*$^{-/-}$*; Nog*$^{+/+}$**	6	3.125	12.00%	6.25%	
***Grhl2*$^{-/-}$*; Nog*$^{+/-}$**	6	3.125	12.00%	12.5%	
***Grhl2*$^{-/-}$*; Nog*$^{-/-}$**	5	3.125	10.00%	6.25%	
	**50**				

C

E12.5

Genotype	Obs	Exp	Obs%	Exp%	χ^2
***Grhl2*$^{+/+}$*; Nog*$^{+/+}$**	6	3.125	8.7%	6.25%	0.0134
***Grhl2*$^{+/+}$*; Nog*$^{+/-}$**	11	6.25	15.94%	12.5%	
***Grhl2*$^{+/+}$*; Nog*$^{-/-}$**	2	3.125	2.9%	6.25%	
***Grhl2*$^{+/-}$*; Nog*$^{+/+}$**	10	6.25	14.49%	12.5%	
***Grhl2*$^{+/-}$*; Nog*$^{+/-}$**	17	12.5	24.64%	25%	
***Grhl2*$^{+/-}$*; Nog*$^{-/-}$**	10	6.25	14.49%	12.5%	
***Grhl2*$^{-/-}$*; Nog*$^{+/+}$**	0	3.125	0%	6.25%	
***Grhl2*$^{-/-}$*; Nog*$^{+/-}$**	5	3.125	7.25%	12.5%	
***Grhl2*$^{-/-}$*; Nog*$^{-/-}$**	0	3.125	0%	6.25%	
	**61**				

D

E14.5

Genotype	Obs	Exp	Obs%	Exp%	χ^2
***Grhl2*$^{+/+}$*; Nog*$^{+/+}$**	2	3.125	6.90%	6.25%	0.0038
***Grhl2*$^{+/+}$*; Nog*$^{+/-}$**	6	6.25	20.69%	12.5%	
***Grhl2*$^{+/+}$*; Nog*$^{-/-}$**	1	3.125	3.45%	6.25%	
***Grhl2*$^{+/-}$*; Nog*$^{+/+}$**	2	6.25	6.90%	12.5%	
***Grhl2*$^{+/-}$*; Nog*$^{+/-}$**	16	12.5	55.17%	25%	
***Grhl2*$^{+/-}$*; Nog*$^{-/-}$**	1	6.25	3.45%	12.5%	
***Grhl2*$^{-/-}$*; Nog*$^{+/+}$**	0	3.125	0%	6.25%	
***Grhl2*$^{-/-}$*; Nog*$^{+/-}$**	0	3.125	0%	12.5%	
***Grhl2*$^{-/-}$*; Nog*$^{-/-}$**	0	3.125	0%	6.25%	
	**28**				

Fig. S4. ***Grhl2*$^{+/-}$*;Nog*$^{+/-}$ intercrosses show non-Mendelian ratios of embryos from E12.5** (A) Diagram showing parental and progeny genotypes present following *Grhl2/Nog* double heterozygous intercross. (B) Mouse genotypes at E10.5. All genotypes were present at approximately Mendelian ratios, indicating that this cross does not lead to embryonic lethality of any progeny, regardless of genotype, before E10.5. (C-D) Mouse genotypes at E12.5 (C) and E14.5 (D) showing a departure from Mendelian ratios and a reduction in the observed numbers of embryos with the *Grhl2*$^{-/-}$ phenotype, due to embryonic lethality of *Grhl2*$^{-/-}$ embryos by E11.5. Viable *Grhl2*$^{-/-}$*;Nog*$^{+/-}$ pups were observed until E12.5, which is one full embryonic day longer than the maximum gestational age survival previously published for *Grhl2*$^{-/-}$ embryos.

Development • Supplementary information

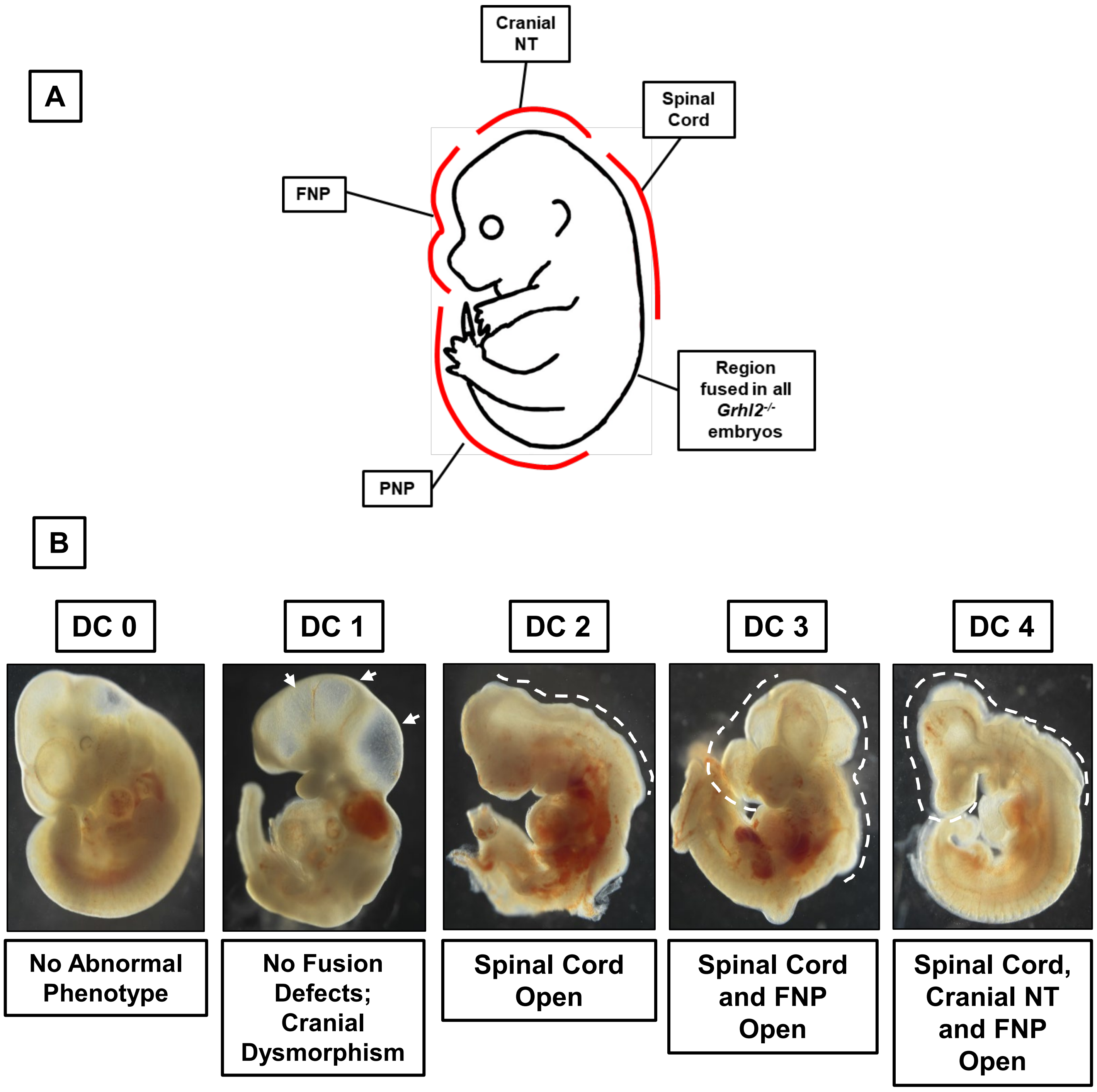

Fig. S5. Regions of frontonasal prominence, cranial neural tube and spinal cord fusion in rescued *Grhl2*$^{-/-}$*;Nog*$^{+/-}$ embryos

(A) Schematic diagram showing regions of the developing frontonasal prominence (FNP), cranial neural tube (NT), spinal cord, and posterior neuropore (PNP) that always remain unfused in *Grhl2*$^{-/-}$ embryos. These embryos do not present with craniorachischisis, as a region of the thoracic spinal cord is always fused. (B) An example of defect classes seen in progeny of *Grhl2*$^{+/-}$*;Nog*$^{+/-}$ intercrosses. DC0 (no abnormal phenotype), DC1 (Cranial Dysmorphism – Spinal cord, FNP and NT closed), DC2 (Spinal cord open, FNP and cranial NT closed), DC3 (Spinal cord open, FNP open, cranial NT closed) and DC4 (Spinal cord open, FNP open, cranial NT open). WT embryos appear as DC0. *Grhl2*$^{-/-}$*;Nog*$^{+/-}$ embryos show evidence of rescue (evidenced by the appearance of embryos with DC1-3) unlike *Grhl2*$^{-/-}$ embryos, which all appear as DC4.

Development • Supplementary information

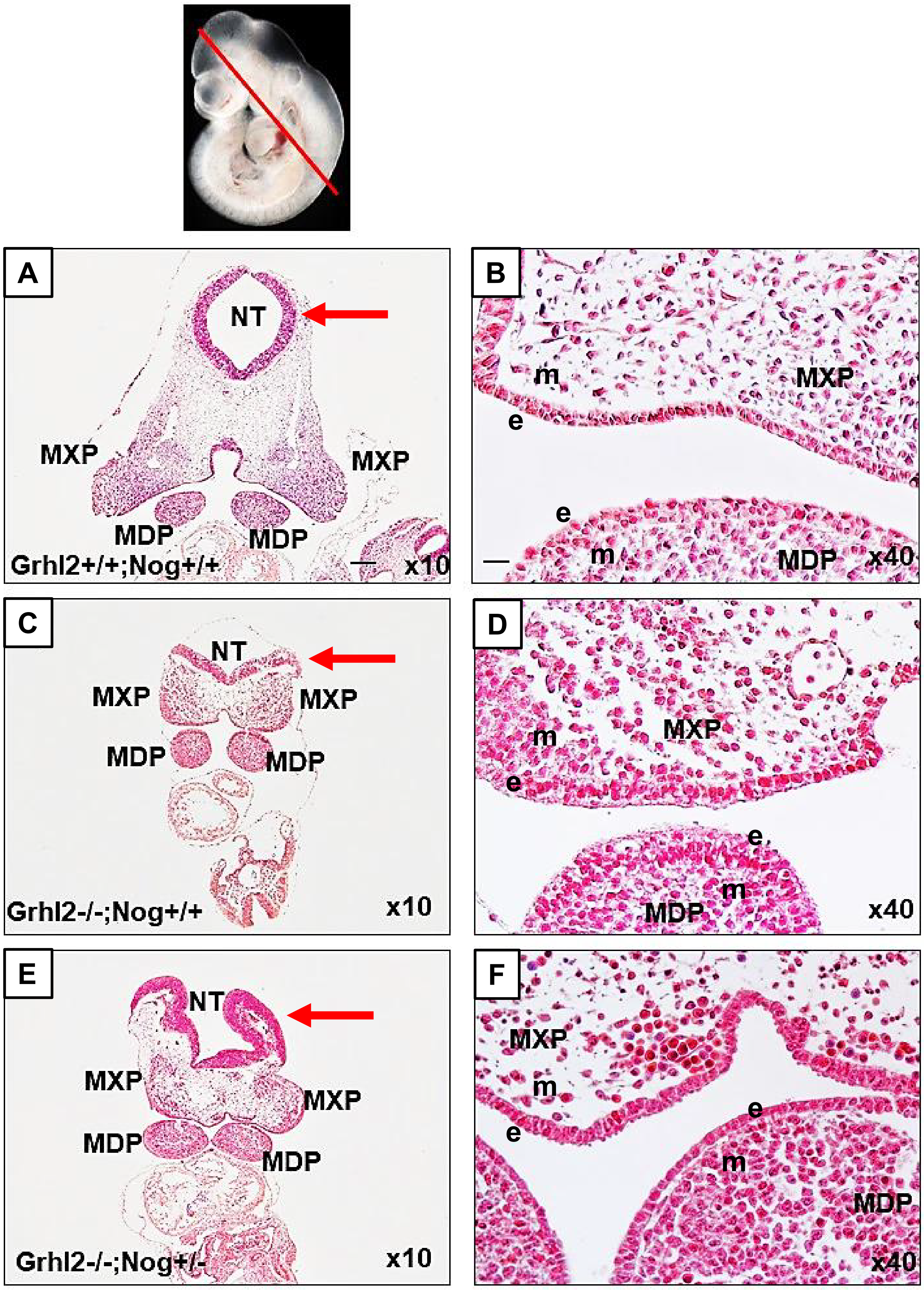

Fig. S6. Partial restoration of NT folding and epithelial layer integrity in E10.5 *Grhl2$^{-/-}$;Nog$^{+/-}$* embryos. (A-B) H&E coronal sections of E10.5 control (*Grhl2$^{+/+}$;Noggin$^{+/+}$*) embryos, showing normal elevation and folding of the NT (red arrow; A) and a clear distinction between epithelial (e) and mesenchymal (m) cells of both the maxillary (MXP) and mandibular (MDP) prominences. In contrast, *Grhl2$^{-/-}$;Nog$^{+/+}$* E10.5 embryos (C-D) present with a flat, non-folded NT (red arrow; C) and a substantially thickened epithelial layer in both MXP and MDP tissue. Both the NT elevation (red arrow; E) and epithelial cellularity are substantially rescued in *Grhl2$^{-/-}$;Nog$^{+/-}$* embryos (E-F). MXP: Maxillary Process, MDP: Mandibular Process, NT: NT, e: epithelial cell layer, m: mesenchymal cells. Scale bar = 200µm (A,C,E), 40µm (B,D,F).

Development • Supplementary information

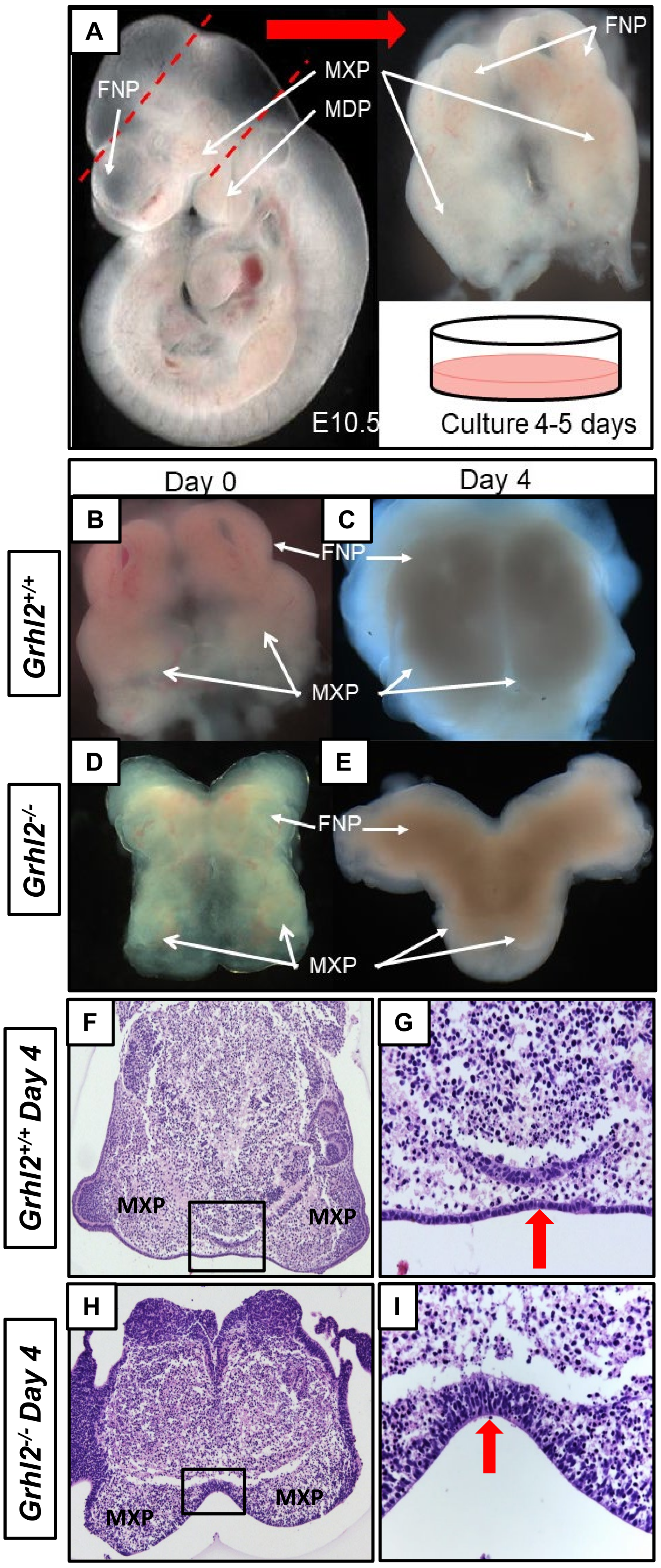

Fig. S7. Pre-palatal explants from WT and *Grhl2*$^{-/-}$ embryos. (A) E10.5 tissue comprising the fronto-nasal prominence (FNP) and maxillary prominence (MXP) was dissected (dotted red lines) to ensure the tissue is extracted with as little mandibular prominence (MDP) or neural tissue as possible. This explant was cultured (free-floating) in supplemented DMEM culture media for 4-5 days. (B-E) Representative images of *Grhl2*$^{+/+}$ (B-C) and *Grhl2*$^{-/-}$ (D-E) tissue (inferior/oral view) immediately after dissection (B, D; Day 0) and after 4 days in culture (C, C; Day 4). (F-I) Coronal sections of *Grhl2*$^{+/+}$ (F-G) and *Grhl2*$^{-/-}$ (G-I) explants following 4 days culture. *Grhl2*$^{+/+}$ tissue presents with well-organised epithelial layers (arrow, G) with a clear demarcation between epithelial and mesenchymal tissue. Conversely, *Grhl2*$^{-/-}$ tissue displayed a thickened epithelial layer (arrow, I), with a poorly defined epithelial-mesenchyme boundary, with epithelial cells showing similar altered cell morphology to that seen *in vivo*. FNP: Frontonasal process MXP: Maxillary process.

Development • Supplementary information

Table S1. ***Noggin*** **gene dosage reduction in** ***Grhl2*** $^{-/-}$ **mice induces a shift in** ***Grhl2*** $^{-/-}$***;Nog*** $^{+/-}$ **mice to less severe phenotypes.** (A) *Grhl2* $^{+/-}$*;Noggin* $^{+/-}$ intercross progeny were characterised based on their phenotypic presentation relating to the fully penetrant split-face phenotype typical *Grhl2* $^{-/-}$ mice. DC0 (Defect class 0) represents no NT or craniofacial defects, reflecting a WT appearance. DC1 includes cranial dysmorphisms that are unrelated to the split-face NT fusion failure phenotypes. DC2 includes mice that display an open NT from the midbrain through to mid-thoracic region, with the FNP and forebrain showing closure. DC3 includes mice that show only the forebrain region of the NT closed with the FNP and the mid-brain to mid-thoracic region open. DC4 includes mice that display complete split-face fusion failure typical of *Grhl2* $^{-/-}$ mice in which fusion failure extends from the FNP to the mid-thoracic closure point. (B) Phenotypic/defect class distribution of each genotype at E10.5 and E12.5, shown as observed number and percent total of that genotype. All *Grhl2* $^{-/-}$*;Nog* $^{+/+}$ mice exhibited the most severe (DC4) NT defect while the majority of *Grhl2* $^{-/-}$*;Nog* $^{+/-}$ mice (~60%) presented with less severe observed phenotypes (defect classes).

A

Defect Class (DC)	Phenotype
DC 0	No abnormal phenotype
DC 1	Cranial Dysmorphism – Spinal cord, FNP and NT closed
DC 2	Spinal cord open, FNP and cranial NT closed
DC 3	Spinal cord open, FNP open, cranial NT closed
DC 4	Spinal cord open, FNP open, cranial NT open

B

E10.5 - 12.5	DC 0	DC 1	DC 2	DC 3	DC 4	TOTAL
Grhl2 $^{+/+}$***; Nog*** $^{+/+}$	8 (100%)	-	-	-	-	**8**
Grhl2 $^{+/+}$***; Nog*** $^{+/-}$	10 (100%)	-	-	-	-	**19**
Grhl2 $^{+/+}$***; Nog*** $^{-/-}$	-	3 (100%)	-	-	-	**3**
Grhl2 $^{+/-}$***; Nog*** $^{+/+}$	16 (100%)	-	-	-	-	**16**
Grhl2 $^{+/-}$***; Nog*** $^{+/-}$	28 (93.3%)	1 (3.3%)	-	-	1 (3.3%)	**30**
Grhl2 $^{+/-}$***; Nog*** $^{-/-}$	1 (7.7%)	12 (92.3%)	-	-	-	**13**
Grhl2 $^{-/-}$***; Nog*** $^{+/+}$	-	-	-	-	6 (100%)	**6**
Grhl2 $^{-/-}$***; Nog*** $^{+/-}$	-	1 (9%)	3 (27.3%)	3 (27.3%)	4 (26.4%)	**11**
Grhl2 $^{-/-}$***; Nog*** $^{-/-}$	-	-	-	1 (20%)	4 (80%)	**5**
TOTAL						**111**

Development • Supplementary information
