# Supplementary material for: Grainyhead-like 2 interacts with noggin to regulate tissue fusion in mouse
Source: Development. 2024 Feb 28;151(5):dev202420. doi: 10.1242/dev.202420 (PMC10946436; doi:10.1242/dev.202420)
Supplement: Supplementary file 1 [file develop-151-202420_review_history.pdf]

Grainyhead-like 2 interacts with noggin to regulate tissue fusion in mouse

Michael E. de Vries, Marina R. Carpinelli, Jarrad N. Fuller, Yindi Sutton, Darren D. Partridge, Alana Auden, Peter J. Anderson, Stephen M. Jane and Sebastian Dworkin
DOI: 10.1242/dev.202420

Editor: Liz Robertson

Review timeline

Original submission:	12 October 2023
Editorial decision:	2 January 2024
First revision received:	23 January 2024
Accepted:	29 January 2024

Original submission

First decision letter

MS ID#: DEVELOP/2023/202420

MS TITLE: Grainyhead-like 2 (Grhl2) interacts with Noggin to regulate tissue fusion in mouse

AUTHORS: Michael de Vries, Marina R Carpinelli, Jarrad N Fuller, Yindi Sutton, Darren D Partridge, Alana Auden, Peter J Anderson, Stephen M Jane, and Sebastian Dworkin

I have now received all the referees reports on the above manuscript, and have reached a decision. The referees' comments are appended below, or you can access them online: please go to BenchPress and click on the 'Manuscripts with Decisions' queue in the Author Area.

The overall evaluation is positive and we would like to publish a revised manuscript in Development, provided that the referees' comments can be satisfactorily addressed. Please attend to all of the reviewers' comments in your revised manuscript and detail them in your point-by-point response. If you do not agree with any of their criticisms or suggestions explain clearly why this is so. If it would be helpful, you are welcome to contact us to discuss your revision in greater detail. Please send us a point-by-point response indicating your plans for addressing the referees' comments, and we will look over this and provide further guidance.

Reviewer 1

Advance summary and potential significance to field

This paper is an interesting study of the modifying effect of Noggin loss of function on the craniofacial phenotype in Grhl2 null embryos. A role for Noggin is elegantly identified by looking at the intersection between genes misregulated in Grhl2 null embryos and genes with conserved GRHL binding sites. Noggin over-expression in Grhl2 null embryos is convincingly demonstrated. Moreover, the data determining the cell proliferation abnormality in Grhl2 epithelia covering branchial arches is well done and convincing. Overall, this paper makes a significant, novel contribution to our understanding of how severe craniofacial phenotypes arise in Grhl2 mutant mice.

Comments for the author

© 2024. Published by The Company of Biologists under the terms of the Creative Commons Attribution License (https://creativecommons.org/licenses/by/4.0/).

The main functional part of the paper is analysis of double Grhl2/Noggin mutants, and here the data are somewhat less clear. The authors conclude their double mutant study by saying: “Taken together, our data support the hypothesis that normalising Nog expression in epithelia can rescue some Grhl2-dependent defects, and identify a novel genetic interaction in epithelial-tissue fusion” (lines 150-152). Why ‘normalising’ in this sentence? They have done the opposite and reduced Nog expression, with some rescue effect.

In fact, the severe split face defect in Grhl2-/-; Nog-/- embryos is hardly rescued at all (only in 1/5 embryos). At first sight, this should be the most striking rescue, according to the hypothesis that Grhl2 null mutants develop abnormally in part by over-expressing Noggin. I find it hard to understand the authors’ explanation but it seems they are suggesting a counteracting effect of Noggin loss in the mesenchyme which, when total (-/-) contributes to split face but when partial (+/-) does not, and so the rescue effect of 50% Nog loss can then be observed. If this is actually the argument, then I suggest the authors make it more explicitly. They should also comment on the finding that Nog null embryos do not exhibit split face, which seems to argue against this explanation for lack of rescue in double homozygotes. Presumably they would predict that epithelium-specific loss of Noggin function would provide full rescue of Grhl2 craniofacial defects?

Status of cranial neural tube closure. I have concerns about how the variable cranial closure phenotypes are reported. While the side-view images in Fig 2A-C are clear (e.g. forebrain closed in C compared with open in B), the images in Fig 2D-I are much less easy to interpret. The view is described in line 572 as “caudal orientation”, but this suggests looking from the back of the embryo whereas the view is actually from the top of the head. Please rephrase. Then, in lines 574-575, the statement about neural tube closure in Grhl2-/-;Nog+/- embryos is unclear. Fig 2F shows an open brain but the legend says other embryos are closed “at the approximate level of the forebrain”. Needs clarification.

It is important to report the range of phenotypes (and their frequencies) seen in Grhl2 mutants compared with Grhl2/Nog double mutants. I found Table S1 very helpful in showing the frequencies of different phenotypic classes among the genotypes. In fact, it was much more helpful than the SEM images in Fig 2.
However, I do not think the Defect Classes (DC) 0-4 are described adequately.
They need accompanying SEM images and/or diagrams to show the appearance of a typical example of each class. Also please specify the exact situation for the FNP, fore-, mid- and hind-brain and spinal cord in each class or sub-class (i.e. the main open/closed combinations of these). What does ‘cranial dysmorphism’ (DC1) mean?

A further comment on Table S1 – the two starting genotypes (Grhl2+/+ and Ghrl2-/-) should be shown separately from the 9 different genotypes that are generated by the double het matings, to allow for possible genetic background effects, once the Nog mutation is introduced.

Methods for pSmad5+ quantitation (Fig 1H) and Ki67 quantitation (Fig 3E) do not appear to be described, and need to be added to the Methods section. Both measures are shown as % positive cells, but what is the denominator – all cells in a particular region? How were counts achieved - manually or using specific software? How many sections/cells were counted per biological replicate sample?

pSmad-1/5/9 expression. Fig S2 shows a detailed analysis by western blot, with quantitation, and I suggest that Fig 1E is therefore removed, as it is not required. Moreover, the description that pSmad-1/5/9 is ‘marginally’ decreased in Grhl2 mutants (lines 554-555) inadequate.

E-cadherin data in Fig 3. This is not well shown, and would benefit from high magnification images of the epithelial layers in Fig 3H,I,J, as is done for Ki67 in Fig 3A’,B’.

Other specific points:

Line 126-128. This sentence is incomplete.
Line 160. Should read: “The thickening of the outermost MXP layer in Grhl2-/- embryos in vivo …”

© 2024. Published by The Company of Biologists under the terms of the Creative Commons Attribution License (https://creativecommons.org/licenses/by/4.0/).

Line 556. Should read: "(F,G) pSmad localisation ….."
Line 560. Should read: "Zone 2: 1-5 cells' depth of mesenchyme extending from BM …."
Lines 587-588. Please clarify that this statement refers to WT embryos.
Line 591. What would constitute "substantial" apoptosis? This sort of description is not adequate.
Fig 1C. It would be helpful if this graph can be labelled as Noggin expression in the same way as for part B.
Fig 3E. This is wrongly mentioned in the legend to show vimentin expression.
Please adjust labelling of parts in Fig 3.
Fig S4. In the legend please comment on the finding that overall Obs and Exp values differ significantly at E12.5 and E14.5 (but not E10.5). The main reasons for this departure from Mendelian ratios should also be given, as it is not immediately obvious from the quite complex data.

Reviewer 2

Advance summary and potential significance to field

This is a very clear report on genetic interactions of the grainyhead transcription and Noggin function in the closure of the neural tube, and in the sizes of branchial arch derivatives.
The work is clearly presented, nicely documented, and very compelling. As an example of a genetic interaction in a mutant line of mice this is a valuable addition to our understanding of face formation and neural tube closure, and as such I find it would be of high interest to readers of Development.

Comments for the author

Looks good to me! Maybe label a few other landmarks in the scanning EM panels which show the Frontonasal process, but it might be nice to know where the midbrain and hindbrain boundaries are in the figures to give context

Also I apologize to the authors for the slow review

First revision

Author response to reviewers' comments

Reviewer 1 Advance Summary and Potential Significance to Field:

This paper is an interesting study of the modifying effect of Noggin loss of function on the craniofacial phenotype in Grhl2 null embryos. A role for Noggin is elegantly identified by looking at the intersection between genes misregulated in Grhl2 null embryos and genes with conserved GRHL binding sites. Noggin over- expression in Grhl2 null embryos is convincingly demonstrated. Moreover, the data determining the cell proliferation abnormality in Grhl2 epithelia covering branchial arches is well done and convincing. Overall, this paper makes a significant, novel contribution to our understanding of how severe craniofacial phenotypes arise in Grhl2 mutant mice.

We thank the reviewer for their very thorough and overall positive assessment of the quality and significance of our work. We have adopted virtually all their suggestions for improvements, as outlined below.

Reviewer 1 Comments for the Author:

"The main functional part of the paper is analysis of double Grhl2/Noggin mutants, and here the data are somewhat less clear. The authors conclude their double mutant study

© 2024. Published by The Company of Biologists under the terms of the Creative Commons Attribution License (https://creativecommons.org/licenses/by/4.0/).

by saying: "Taken together, our data support the hypothesis that normalising Nog expression in epithelia can rescue some Grhl2-dependent defects, and identify a novel genetic interaction in epithelial-tissue fusion" (lines 150-152). Why 'normalising' in this sentence? They have done the opposite and reduced Nog expression, with some rescue effect."

We originally used the term "normalising" to indicate that the overall increase in Noggin expression seen in *Grhl2*$^{-/-}$ embryos (Fig. 1B) and within the MXP epithelium (Fig. 1C) is reduced when these embryos are on a *Nog*$^{+/-}$ background. We felt that using the term "reduced" in this context may have led readers to mistakenly assume that *Nog* expression is reduced below wild-type levels. However, we have now amended the term "normalising Noggin expression" to "reducing elevated Noggin expression", which we agree better represents our findings.

"In fact, the severe split face defect in Grhl2-/-; Nog-/- embryos is hardly rescued at all (only in 1/5 embryos). At first sight, this should be the most striking rescue, according to the hypothesis that Grhl2 null mutants develop abnormally in part by over-expressing Noggin. I find it hard to understand the authors' explanation but it seems they are suggesting a counteracting effect of Noggin loss in the mesenchyme which, when total (-/-) contributes to split face, but when partial (+/-) does not, and so the rescue effect of 50% Nog loss can then be observed. If this is actually the argument, then I suggest the authors make it more explicitly.

Initially, we also thought that the most striking rescue should be seen in *Nog*$^{-/-}$;*Grhl2*$^{-/-}$ double mutants. However this is certainly complicated by the fact that *Noggin* is expressed within the NCC-derived mesenchymal layer, whereas *Grhl2* is not. Moreover, loss of *Grhl2* in the NCC also does not lead to any phenotypes, whereas loss of *Noggin* only within the NCC does lead to substantial craniofacial defects, including cleft palate. Hence, we believe that we do not see substantial rescue as the phenotypes would be additive (i.e. comprising both NCC-specific, and epithelium-specific defects).

Our data suggest that at least one allele of *Noggin* is needed (i.e. 50% gene dosage), functioning within the epithelium, to establish or maintain the BMP-pSMAD gradients within the mesenchyme. This principle is also likely in the context of neurulation, where *Grhl2* is only expressed in the non-neural ectoderm overlying the neural tube.

We have therefore reworded this section as follows:

"*Grhl2*$^{-/-}$;*Nog*$^{-/-}$ embryos did not present with substantial rescue of *Grhl2*-dependent phenotypes in any of the FNP, cranial NT or spinal cord (4/5, 80%, as DC4). *Noggin*-null embryos present with defects of cranial neural tube fusion, manifesting as exencephaly (McMahon et al., 1998; Tylzanowski et al., 2006). Loss of *Noggin* exclusively within the neural crest also results in craniofacial defects, including clefting (Graf et al., 2016; He et al., 2010; Matsui and Klingensmith, 2014; McMahon et al., 1998), suggesting that one reason for lack of rescue in *Grhl2*$^{-/-}$;*Nog*$^{-/-}$ embryos may be due to additive mesenchyme-specific defects as a consequence of full *Nog* loss, irrespective of *Grhl2* involvement. Additionally, given that overexpression of *Nog* in palatal mesenchyme expression also leads to tissue fusion defects (Li et al., 2021), it is clear that tightly-regulated homeostatic expression of *Nog* is essential for correct tissue fusion in development. Therefore, our data suggest that at least one allele of *Nog* (50% gene dosage) is required to establish, or maintain, homeostatic BMP-pSMAD gradients within both the developing neuroectoderm and palatal primordia in *Grhl2*$^{-/-}$ embryos.

They should also comment on the finding that Nog null embryos do not exhibit split face, which seems to argue against this explanation for lack of rescue in double homozygotes."

Our wording that we expected complete Noggin loss to rescue the "split-face" defect of *Grhl2*- loss was not quite correct, because as the reviewer quite rightly points out, *Noggin*-null mutants do not present with a split-face phenotype. However they do present with an open cranial NT phenotype (McMahon et al., 1998; Tylzanowski et al., 2006), which appears to arise from ~E14.5, but manifests as cranial dysmorphism at earlier timepoints (E10.5-E12.5). It is also possible that significantly greater fusion rescue of both the NT and craniofacial region would be seen in *Grhl2*$^{-/-}$

© 2024. Published by The Company of Biologists under the terms of the Creative Commons Attribution License (https://creativecommons.org/licenses/by/4.0/).

;*Nog*$^{+/-}$ embryos if the E11.5 lethality in *Grhl2*$^{-/-}$embryos could be overcome.

We have therefore changed the wording to indicate rescue of cranial NT closure at E10.5-E12.5, rather than split-face.

Presumably they would predict that epithelium-specific loss of Noggin function would provide full rescue of Grhl2 craniofacial defects?"

Yes, we certainly do believe that we would see far more complete rescue in the above scenario. However, as *Grhl2* is a transcription factor, it is certain to regulate multiple other genes within the palatal epithelium, in addition to *Noggin*, in mediating epithelial stability (and consequently craniofacial rescue) in *Grhl2*$^{-/-}$ embryos.

As we show in Fig. S1, we already have a list of 20 other putative target genes that may form part of this network within the craniofacial primordia (including some known epithelial genes, e.g. *Ppl, Pvrl4, Cldn4* and *Cdh1*). Moreover, numerous experimentally-validated genes that operate within the *Grhl2/Grhl3* genetic network, including *IRF6, GSK3B, FGF8, p63, Tfap2a* and *PTEN* are known to be involved in palatal development and fusion, and could therefore also comprise additional mechanisms.

For the sake of clarity, we have chosen to focus on extensive discussion of these other putative pathways within the present study.

"Status of cranial neural tube closure. I have concerns about how the variable cranial closure phenotypes are reported. While the side-view images in Fig 2A-C are clear (e.g. forebrain closed in C compared with open in B), the images in Fig 2D-I are much less easy to interpret. The view is described in line 572 as "caudal orientation", but this suggests looking from the back of the embryo, whereas the view is actually from the top of the head. Please rephrase."

Our apologies for this inadvertent error – Panels D-F are indeed in dorsal view, whereas G-I are caudal. This has been corrected in the figure legend.

"Then, in lines 574-575, the statement about neural tube closure in Grhl2-/-;Nog+/- embryos is unclear. Fig 2F shows an open brain but the legend says other embryos are closed "at the approximate level of the forebrain". Needs clarification."

We agree that this should have been presented more clearly. We have updated the phenotypic incidence and highlighted regions of NT closure in Fig. 2. We have also improved the description clarity of our "defect classes" in the text, figure legends and supplementary data, and have included a new supplementary figure (Fig. S5) to better highlight regions of the FNP/NT that remain open in rescued embryos. We have also clarified our terminology to be consistent when describing both the SEM images and the defect classes.

"It is important to report the range of phenotypes (and their frequencies) seen in Grhl2 mutants compared with Grhl2/Nog double mutants. I found Table S1 very helpful in showing the frequencies of different phenotypic classes among the genotypes. In fact, it was much more helpful than the SEM images in Fig 2.

However, I do not think the Defect Classes (DC) 0-4 are described adequately. They need accompanying SEM images and/or diagrams to show the appearance of a typical example of each class. Also please specify the exact situation for the FNP, fore-, mid- and hind-brain and spinal cord in each class or sub-class (i.e. the main open/closed combinations of these). "

We agree that such a figure and clearer descriptions would greatly enhance our manuscript. We have therefore included a new supplementary figure (Fig. S5) that shows a schematic of a mouse embryo with the FNP, Cranial NT and spinal cord labelled, alongside a typical example of each defect class. We believe that this, coupled with our clearer, consistent descriptions (as noted above) helps to clarify the regions of fusion/non-fusion (and combinations of those) across our different embryo genotypes.

© 2024. Published by The Company of Biologists under the terms of the Creative Commons Attribution License (https://creativecommons.org/licenses/by/4.0/).

“What does ‘cranial dysmorphism’ (DC1) mean?”

In this context, cranial dysmorphism refers to any deviation of craniofacial development (away from WT) in the absence of fusion defects of the FNP, cranial NT or spinal cord. This may include phenotypes consistent with hydrocephalus (as in Fig. S5), defects in eye formation, or aberrations in the size/shape of the developing head, and has been shown qualitatively previously for *Noggin*-null embryos.

“A further comment on Table S1 - the two starting genotypes (Grhl2+/+ and Ghrl2-/-) should be shown separately from the 9 different genotypes that are generated by the double het matings, to allow for possible genetic background effects, once the Nog mutation is introduced.”

To clarify, we have actually maintained *Grhl2*$^{+/-}$;*Nog*$^{+/-}$ adults of both sexes at our institution for over 7 years now, which encompasses >15 breeding generations, hence the genetic background for all embryos is identical. We have amended the ”Animals” section in the methods to indicate this. The phenotypes of both *Grhl2*$^{+/+}$ and *Grhl2*$^{-/-}$ embryos of the *Grhl2*$^{tm1.1Jane}$ strain have been documented elsewhere (Rifat et al., 2010), however we have now amended Table S1 to make it easier to read, with clearer groupings of genotypes.

“Methods for pSmad5+ quantitation (Fig 1H) and Ki67 quantitation (Fig 3E) do not appear to be described, and need to be added to the Methods section. Both measures are shown as % positive cells, but what is the denominator - all cells in a particular region? How were counts achieved - manually or using specific software? How many sections/cells were counted per biological replicate sample?”

Our apologies for this oversight. All cell counts for both pSMAD5+ and Ki67 quantitation were done manually. For pSMAD5, both pSMAD5^{+} and pSMAD5^{-} cells within each zone outlined in Fig. 1E-F were counted (within all MXP tissue, one side, visible in each section), from n = 3 biological replicates per genotype. For Ki67 quantitation, three independent fields were selected per section (slightly larger in size than the boxed regions shown in Fig. 3A-B), comprising both the epithelial region and adjoining mesenchyme, and all Ki67^{+} and Ki67^{-} cells were counted. These counts were also performed from n = 3 biological replicates per genotype.

This information has been added to the methods section, as requested.

“pSmad-1/5/9 expression. Fig S2 shows a detailed analysis by western blot, with quantitation, and I suggest that Fig 1E is therefore removed, as it is not required.”

This has been done as requested.

“Moreover, the description that pSmad-1/5/9 is ‘marginally’ decreased in Grhl2 mutants (lines 554-555) inadequate.”

We agree – the term “marginally” has been replaced with “not significantly”.

“E-cadherin data in Fig 3. This is not well shown, and would benefit from high magnification images of the epithelial layers in Fig 3H,I,J, as is done for Ki67 in Fig 3A’,B’.”

This has been done as suggested, included as three new panels (H’-J’) in Fig. 3.

Other specific points:

“Line 126-128. This sentence is incomplete.

This has been corrected.

Line 160. Should read: “The thickening of the outermost MXP layer in Grhl2-/- embryos in vivo …”

© 2024. Published by The Company of Biologists under the terms of the Creative Commons Attribution License (https://creativecommons.org/licenses/by/4.0/).

This has been corrected as suggested.

Line 556. Should read: "(F,G) pSmad localisation ….."

This has been corrected as suggested.

Line 560. Should read: "Zone 2: 1-5 cells' depth of mesenchyme extending from BM …."

This has been corrected as suggested.

Lines 587-588. Please clarify that this statement refers to WT embryos.

This has been corrected as suggested.

Line 591. What would constitute "substantial" apoptosis? This sort of description is not adequate.

We agree – the term "substantial" has been removed.

Fig 1C. It would be helpful if this graph can be labelled as Noggin expression, in the same way as for part B.

This has been corrected as suggested.

Fig 3E. This is wrongly mentioned in the legend to show vimentin expression. Please adjust labelling of parts in Fig 3.

Thank you for picking this error up - the labelling of panels in Figure 3 has now been corrected.

Fig S4. In the legend please comment on the finding that overall Obs and Exp values differ significantly at E12.5 and E14.5 (but not E10.5). The main reasons for this departure from Mendelian ratios should also be given, as it is not immediately obvious from the quite complex data."

The reason for this is that *Grhl2*$^{-/-}$ embryos are known to be lethal by E11.5 (Carpinelli et al., 2020; Rifat et al., 2010), hence these embryos are not present at later timepoints. This explanation has been added to the legend as requested, and the sentence amended as follows:

"(C-D) Mouse genotypes at E12.5 (C) and E14.5 (D) showing a departure from Mendelian ratios and a reduction in the observed numbers of embryos with the *Grhl2*$^{-/-}$ phenotype, due to embryonic lethality of *Grhl2*$^{-/-}$ embryos by E11.5. Viable *Grhl2*$^{-/-}$;*Nog*$^{+/-}$ pups were observed until E12.5, which is one full embryonic day longer than the maximum gestational age survival previously published for *Grhl2*$^{-/-}$ embryos."

This has also been clarified in the main body of the text.

******* Reviewer 2 Advance Summary and Potential Significance to Field: This is a very clear report on genetic interactions of the grainyhead transcription and Noggin function in the closure of the neural tube, and in the sizes of branchial arch derivatives.**
The work is clearly presented, nicely documented, and very compelling. As an example of a genetic interaction in a mutant line of mice this is a valuable addition to our understanding of face formation and neural tube closure, and as such I find it would be of high interest to readers of Development.

We thank the reviewer for their positive evaluation of our work.

Reviewer 2 Comments for the Author:

© 2024. Published by The Company of Biologists under the terms of the Creative Commons Attribution License (https://creativecommons.org/licenses/by/4.0/).

Looks good to me! Maybe label a few other landmarks in the scanning EM panels which show the Frontonasal process, but it might be nice to know where the midbrain and hindbrain boundaries are in the figures to give context

We have included a label for the approximate location for the future midbrain-hindbrain boundary (MHB) to better orient the reader. Additionally, we have now also added a new supplementary figure (new Fig. S5, as per response to Reviewer #1), which we believe brings much greater clarity to the precise anatomical locations of rescued fusion sites in *Grhl2*$^{-/-}$*;Nog*$^{+/-}$ embryos.

References

Carpinelli, M. R., de Vries, M. E., Auden, A., Butt, T., Deng, Z., Partridge, D. D., Miles, L. B., Georgy, S. R., Haigh, J. J., Darido, C., et al. (2020). Inactivation of Zeb1 in GRHL2- deficient mouse embryos rescues mid-gestation viability and secondary palate closure. *Dis Model Mech* **13**.

Graf, D., Malik, Z., Hayano, S. and Mishina, Y. (2016). Common mechanisms in development and disease: BMP signaling in craniofacial development. *Cytokine Growth Factor Rev* **27**, 129-139.

He, F., Xiong, W., Wang, Y., Matsui, M., Yu, X., Chai, Y., Klingensmith, J. and Chen, Y. (2010). Modulation of BMP signaling by Noggin is required for the maintenance of palatal epithelial integrity during palatogenesis. *Developmental biology* **347**, 109-121.

Li, N., Liu, J., Liu, H., Wang, S., Hu, P., Zhou, H., Xiao, J. and Liu, C. (2021). Altered BMP-Smad4 signaling causes complete cleft palate by disturbing osteogenesis in palatal mesenchyme. *J Mol Histol* **52**, 45-61.

Matsui, M. and Klingensmith, J. (2014). Multiple tissue-specific requirements for the BMP antagonist Noggin in development of the mammalian craniofacial skeleton. *Developmental biology* **392**, 168-181.

McMahon, J. A., Takada, S., Zimmerman, L. B., Fan, C. M., Harland, R. M. and McMahon, A. P. (1998). Noggin-mediated antagonism of BMP signaling is required for growth and patterning of the neural tube and somite. *Genes & development* **12**, 1438-1452.

Rifat, Y., Parekh, V., Wilanowski, T., Hislop, N. R., Auden, A., Ting, S. B., Cunningham, J. M. and Jane, S. M. (2010). Regional neural tube closure defined by the Grainy head-like transcription factors. *Developmental biology* **345**, 237-245.

Tylzanowski, P., Mebis, L. and Luyten, F. P. (2006). The Noggin null mouse phenotype is strain dependent and haploinsufficiency leads to skeletal defects. *Dev Dyn* **235**, 1599-1607.

Second decision letter

MS ID#: DEVELOP/2023/202420

MS TITLE: Grainyhead-like 2 (Grhl2) interacts with Noggin to regulate tissue fusion in mouse

AUTHORS: Michael de Vries, Marina R Carpinelli, Jarrad N Fuller, Yindi Sutton, Darren D Partridge, Alana Auden, Peter J Anderson, Stephen M Jane, and Sebastian Dworkin

ARTICLE TYPE: Research Report

I am happy to tell you that your manuscript has been accepted for publication in Development, pending our standard ethics checks.

© 2024. Published by The Company of Biologists under the terms of the Creative Commons Attribution License (https://creativecommons.org/licenses/by/4.0/).

<u>Reviewer 1</u>

Advance summary and potential significance to field

The authors have worked hard in revising the manuscript, which is much improved as a result. All of my comments and suggestions have been acted upon appropriately and I am pleased to recommend publication of this interesting and important paper in Development.

Comments for the author

No further changes required.

© 2024. Published by The Company of Biologists under the terms of the Creative Commons Attribution License (https://creativecommons.org/licenses/by/4.0/).
